# Zebrafish *mbnl* mutants model physical and molecular phenotypes of myotonic dystrophy

**DOI:** 10.1242/dmm.045773

**Published:** 2021-06-14

**Authors:** Melissa N. Hinman, Jared I. Richardson, Rose A. Sockol, Eliza D. Aronson, Sarah J. Stednitz, Katrina N. Murray, J. Andrew Berglund, Karen Guillemin

**Affiliations:** 1 Institute of Molecular Biology, University of Oregon, Eugene, OR 97403, USA; 2 RNA Institute, State University of New York at Albany, Albany, NY 12222, USA; 3 Department of Biochemistry and Molecular Biology, Center for NeuroGenetics, University of Florida, Gainesville, FL 32611, USA; 4 Institute of Neuroscience, University of Oregon, Eugene, OR 97403, USA; 5 Zebrafish International Resource Center, University of Oregon, Eugene, OR 97403, USA; 6 Humans and the Microbiome Program, CIFAR, Toronto, ON M5G 1M1, Canada

**Keywords:** MBNL1, MBNL2, MBNL3, Myotonic dystrophy, Zebrafish disease models

## Abstract

The muscleblind RNA-binding proteins (MBNL1, MBNL2 and MBNL3) are highly conserved across vertebrates and are important regulators of RNA alternative splicing. Loss of MBNL protein function through sequestration by CUG or CCUG RNA repeats is largely responsible for the phenotypes of the human genetic disorder myotonic dystrophy (DM). We generated the first stable zebrafish (*Danio rerio*) models of DM-associated *MBNL* loss of function through mutation of the three zebrafish *mbnl* genes. In contrast to mouse models, zebrafish double and triple homozygous *mbnl* mutants were viable to adulthood. Zebrafish *mbnl* mutants displayed disease-relevant physical phenotypes including decreased body size and impaired movement. They also exhibited widespread alternative splicing changes, including the misregulation of many DM-relevant exons. Physical and molecular phenotypes were more severe in compound *mbnl* mutants than in single *mbnl* mutants, suggesting partially redundant functions of Mbnl proteins. The high fecundity and larval optical transparency of this complete series of zebrafish *mbnl* mutants will make them useful for studying DM-related phenotypes and how individual Mbnl proteins contribute to them, and for testing potential therapeutics.

This article has an associated First Person interview with the first author of the paper.

## Abstract (precis)

**Summary:** We generated a panel of single, double and triple homozygous zebrafish *mbnl* mutants to model myotonic dystrophy. They exhibited decreased body size, impaired movement and widespread, disease-relevant alternative splicing changes.

## INTRODUCTION

The muscleblind (MBNL) family of RNA-binding proteins (MBNL1, MBNL2 and MBNL3) is highly conserved in structure and function across multicellular species (Oddo et al., 2016). Vertebrate MBNL proteins contain two pairs of zinc finger motifs that bind to consensus YGCY sequences (where Y is a pyrimidine) in target RNAs and regulate multiple aspects of RNA metabolism, including alternative splicing (Ashwal-Fluss et al., 2014; Batra et al., 2014; Goers et al., 2010; Grammatikakis et al., 2011; Ho et al., 2004; Rau et al., 2011; Wang et al., 2012; Warf and Berglund, 2007). In general, MBNL proteins promote exon skipping or inclusion when bound to introns upstream or downstream of an alternative exon, respectively (Du et al., 2010; Goers et al., 2010; Wang et al., 2012). Regulation of alternative splicing by MBNL proteins influences the molecular and biological functions of hundreds of target genes.

**Fig. 1.**
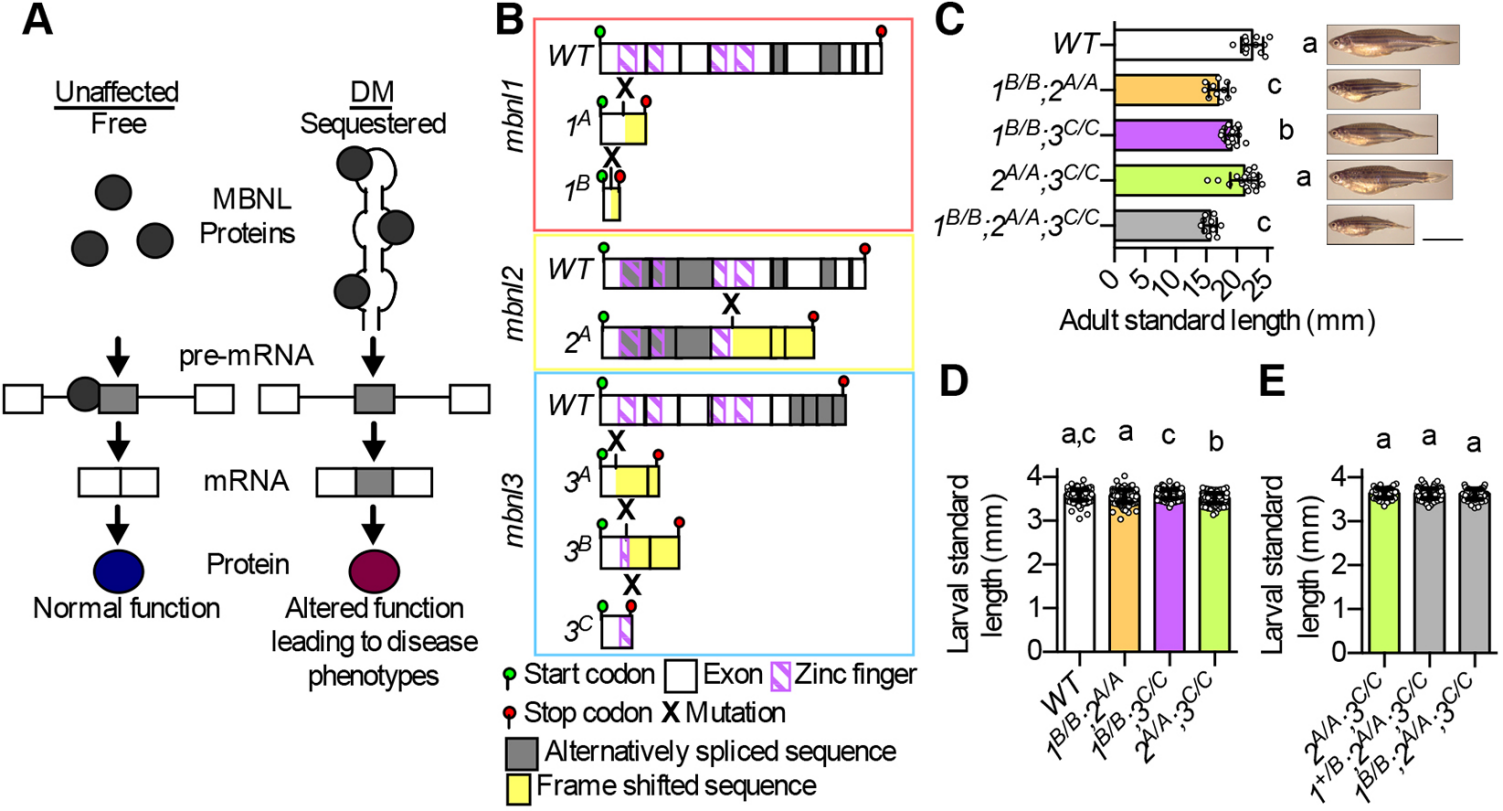
**Mutation of zebrafish *mbnl* genes resulted in decreased adult body size.** (A) Primary molecular mechanism of the human genetic disorder myotonic dystrophy (DM). In unaffected individuals, free MBNL1, MBNL2 and MBNL3 proteins bind to target pre-mRNAs and regulate the inclusion of alternative exons in mRNAs. MBNL proteins suppress alternative exon inclusion in the example shown here, but they promote inclusion of other target alternative exons. In individuals with DM, MBNL proteins are sequestered by long CUG (DM1) or CCUG (DM2) repeat RNAs, which decreases their availability to bind to target pre-mRNAs and alters mRNA isoform production and downstream protein function. (B) Diagram of WT and mutant zebrafish *mbnl1*, *mbnl2* and *mbnl3* predicted coding sequences. *mbnl1* mutant alleles are denoted as *1^A^* and *1^B^*, *mbnl2* alleles as *2^A^*, and *mbnl3* alleles as *3^A^*, *3^B^* and *3^C^*. Mutant sequences are shown in Table S2. (C) Standard length of young adult 60 days post-fertilization (dpf) WT and *mbnl* mutant zebrafish that were raised in the same tank. On the right are representative images of fish of each genotype taken at 76 dpf. Scale bar: 10 mm. (D,E) Standard length of 7 dpf WT and double *mbnl* mutant zebrafish (D) or clutchmates from an incross of *1^+/B^;2^A/A^;3^C/C^* fish (E). In C-E, each dot represents one fish and data are presented as mean±s.d. Data were analyzed by ordinary one-way ANOVA with Tukey's multiple comparisons test. Data bars that do not share the same letter above them are significantly different from one another. Raw data and statistical analysis details are in Table S5.

Myotonic dystrophy (DM) types 1 and 2 (DM1 and DM2) are human genetic disorders caused primarily by MBNL protein loss of function. In DM, expression of expanded CTG or CCTG repeat RNAs leads to the formation of RNA stem loop structures, which contain dozens to thousands of YGCY motifs that sequester MBNL proteins, blocking their normal functions (Brook et al., 1992; Fardaei et al., 2002; Fu et al., 1992; Liquori et al., 2001; Mahadevan et al., 1992; Miller et al., 2000) (Fig. 1A). DM1 disease severity worsens with increasing CTG repeat length, likely due to increased MBNL protein sequestration (Yum et al., 2017). Although best known for its skeletal muscle phenotypes such as weakness, myotonia, atrophy and pain, DM causes severe multi-systemic symptoms including cataracts, breathing difficulties, behavioral and psychological disorders, sleep disorders, insulin resistance, heart conduction abnormalities, cardiomyopathy, and alterations in the motility and microbiota of the gut (Bellini et al., 2006; Hilbert et al., 2017; Tarnopolsky et al., 2010; Thomas et al., 2018; Tieleman et al., 2008; Wenninger et al., 2018). Misregulation of specific alternative splicing events underlies disease phenotypes (Thomas et al., 2018). For example, mis-splicing of *RYR1* and *ATP2A1* contributes to altered calcium homeostasis in DM1 muscle (Kimura et al., 2005; Zhao et al., 2015).

*Mbnl* mutant mice have been invaluable for studying how individual Mbnl proteins contribute to DM-related phenotypes. Mouse *Mbnl1* is widely expressed, while *Mbnl2* expression is brain enriched, and *Mbnl3* is expressed primarily during embryonic development and injury-induced adult skeletal muscle regeneration (Charizanis et al., 2012; Lee et al., 2013; Poulos et al., 2013). *Mbnl1^−/−^* mice exhibit alternative splicing changes, myotonia, myopathy and cataracts, but do not have other DM-related phenotypes such as muscle weakness (Kanadia et al., 2003; Lee et al., 2013). *Mbnl2^−/−^* mice exhibit DM-like central nervous system phenotypes including mis-splicing in the brain, abnormal sleep patterns and spatial memory deficits (Charizanis et al., 2012). *Mbnl3^−/−^* mice exhibit delays in injury-induced muscle regeneration, accelerated onset of age-related pathologies and gene expression changes, but display minimal changes in alternative splicing (Choi et al., 2016; Poulos et al., 2013). Compound *Mbnl* mutant mice have more severe phenotypes than single mutant mice, suggesting that Mbnl proteins have redundant functions. *Mbnl1^−/−^;Mbnl2^−/−^* mice are embryonic lethal, whereas *Mbnl1^−/−^;Mbnl2^+/−^* mice are viable, but have severe mis-splicing, decreased body weight, myotonia, progressive muscle weakness and reduced lifespan (Lee et al., 2013). Muscle-specific double (*Mbnl1/Mbnl2*) and triple (*Mbnl1/Mbnl2/Mbnl3*) homozygous mutant mice are reduced in size, have dramatic and widespread alternative splicing changes, muscle weakness, and most die in the neonatal period due to respiratory distress (Thomas et al., 2017). Compound *Mbnl* mutant mice recapitulate many of the phenotypes associated with severe forms of DM, but their utility in experiments is limited due to difficulty in generating them in large numbers.

We used a complementary vertebrate organism, the zebrafish, to model DM-associated MBNL loss of function. Like mice, zebrafish have a single ortholog of each human *MBNL* gene (Liu et al., 2008). Unlike mice, zebrafish embryos are transparent and develop rapidly and externally, enabling direct studies of developmental phenotypes and of DM-related phenotypes such as altered gut motility and abnormal heart rhythm. In addition, zebrafish produce hundreds of embryos at once, and adults can be maintained more cheaply than mice, enabling experiments with large numbers of animals that improve the statistical power to study subtle or variable phenotypes. Two existing zebrafish DM models have severe limitations. Embryos that are transiently injected with CUG repeat RNA have subtle developmental abnormalities, but do not exhibit alternative splicing changes (Todd et al., 2014). Embryos in which *mbnl2* expression is temporarily knocked down using morpholinos, which often have off-target effects, exhibit profound morphological abnormalities that are inconsistent with the much milder phenotypes observed in *Mbnl2^−/−^* mice (Charizanis et al., 2012; Machuca-Tzili et al., 2011). Neither zebrafish model can be used to study DM-relevant phenotypes beyond early development.

In this study, we made homozygous zebrafish *mbnl1*, *mbnl2* and *mbnl3* loss-of-function mutants, which were crossed to generate double (*mbnl1/mbnl2*, *mbnl2/mbnl3* and *mbnl1/mbnl3*) and triple (*mbnl1/mbnl2/mbnl3*) homozygous mutants, all of which were viable to adulthood. Zebrafish *mbnl* mutants exhibited DM-relevant physical phenotypes including decreased body size and impaired motor function. They also exhibited widespread alternative splicing changes, including many of the same changes that were present in DM patients and mouse *Mbnl* mutants. These alternative splicing changes occurred both in larval and adult fish, but were most dramatic in adult skeletal and heart muscle. As in mice, double and triple homozygous zebrafish *mbnl* mutants exhibited more severe phenotypes than single mutants. Thus, zebrafish *mbnl* mutants had physical and molecular phenotypes consistent with those present in DM, and are powerful new vertebrate models for studies of *MBNL* function.

## RESULTS

### Generation of a comprehensive set of zebrafish *mbnl* mutants

To model DM-associated *MBNL* loss of function, we targeted constitutively included exons in each zebrafish *mbnl* gene using CRISPR, and established two *mbnl1* (*1^A/A^* and *1^B/B^*), one *mbnl2* (*2^A/A^*) and three *mbnl3* (*3^A/A^*, *3^B/B^* and *3^C/C^*) mutant lines (Fig. 1B; Tables S1 and S2) (Liu et al., 2008). Although we lacked antibodies suitable for assessing zebrafish Mbnl protein levels (see Materials and Methods for panel of commercial antibodies tested), each *mbnl* mutation was predicted to result in loss of protein function due to frameshift and/or introduction of an early stop codon, leading to disruption of one or more of the zinc fingers that mediate RNA binding (Fig. 1B; Table S2) (Grammatikakis et al., 2011). Homozygous larval mutants exhibited decreased (*2^A/A^*, *3^A/A^* and *3^B/B^*) or unchanged (*1^A/A^*, *1^B/B^* and *3^C/C^*) levels of the mRNA expressed from the mutated gene, and the expression of the other *mbnl* family members was largely unchanged (Fig. S1A-C), arguing against genetic compensation in these lines (El-Brolosy et al., 2019).

All homozygous *mbnl1*, *mbnl2* and *mbnl3* mutants survived to adulthood in roughly Mendelian ratios (Table S3), were fertile, and did not exhibit the dramatic morphological phenotypes that were previously observed in *mbnl2* morpholino-injected larvae (Machuca-Tzili et al., 2011). Double (*1^B/B^;2^A/A^*, *1^B/B^;3^C/C^* and *2^A/A^;3^C/C^*) and triple (*1^B/B^;2^A/A^;3^C/C^*) mutants were also viable, although triple mutants were present in lower than expected numbers and were unable to produce embryos except through *in vitro* fertilization (Table S3). This was, to our knowledge, the first time that *mbnl1/mbnl3* and *mbnl2/mbnl3* double homozygous mutant animals were generated. The viability of *1^B/B^;2^A/A^* and *1^B/B^;2^A/A^;3^C/C^* zebrafish was in contrast to the neonatal lethality of analogous mouse models (Lee et al., 2013; Thomas et al., 2018, 2017). Our analysis of previously published RNA sequencing (RNA-Seq) data (Mehjabin et al., 2019) indicated that all three *mbnl* mRNAs were present at low or very low levels in wild-type (WT) unfertilized eggs (Fig. S1G), consistent with a previous report that only maternally deposited mRNAs of *mbnl1* and *mbnl2* could be detected by non-quantitative reverse transcription PCR (RT-PCR) (Liu et al., 2008). However, the maternally deposited *mbnl* mRNAs did not account for viability of our mutants, as both zygotic and maternal-zygotic single, double and triple *mbnl* homozygous mutants survived to adulthood. In summary, we created the first complete panel of vertebrate *mbnl1*, *mbnl2* and *mbnl3* single, double and triple homozygous mutants for modeling DM.

### Mutation of *mbnl* genes leads to decreased zebrafish size

We asked whether zebrafish had similar phenotypes to mice, in which compound, but not single, *Mbnl* mutants were significantly smaller than WT (Lee et al., 2013; Thomas et al., 2017). We raised WT, double and triple homozygous *mbnl* mutant fish under identical conditions and measured their lengths in early adulthood. WT fish were significantly larger than *1^B/B^;3^C/C^* fish, which in turn were larger than *1^B/B^;2^A/A^* and *1^B/B^;2^A/A^;3^C/C^* fish (Fig. 1C). Although *2^A/A^;3^C/C^* fish were slightly smaller than WT fish, the difference was not statistically significant (Fig. 1C). Mutation of *mbnl1*, *mbnl2* or *mbnl3* alone was not sufficient to decrease adult zebrafish size (Fig. S2A-C). Taken together, these results suggested that zebrafish Mbnl proteins have partially redundant functions, as the growth defect was more dramatic in compound mutants than in single mutants.

We also measured 7 days post-fertilization (dpf) larval fish prior to exogenous feeding. All double homozygous *mbnl* mutant larvae were similar in size to WT, except for *2^A/A^;3^C/C^*, which were slightly smaller than WT (Fig. 1D). *1^B/B^;2^A/A^;3^C/C^* larvae were similar in size to their *1^+/B^;2^A/A^;3^C/C^* and *2^A/A^;3^C/C^* clutchmates, and all single homozygous larvae were either similar in size or slightly larger than WT (Fig. 1E; Fig. S2D). These results suggest that the reduced size phenotype in zebrafish *mbnl* mutants arose later in development.

Histological analysis of adult skeletal muscle that was performed by a fish pathologist indicated that the fish genotypes with the most profound size phenotypes, *1^B/B^;2^A/A^* and *1^B/B^;2^A/A^;3^C/C^*, did not exhibit the dramatic myofiber atrophy or centralized nuclei that were described in compound *mbnl* mutant mice and in individuals with DM (Fig. S3A) (Thomas et al., 2018, 2017). There was a subtle but significant decrease in the absolute cross-sectional areas of individual muscle fibers in *1^B/B^;2^A/A^* and *1^B/B^;2^A/A^;3^C/C^* fish compared to WT (Fig. S3B). This could be an indication of muscle pathology, but could also be a consequence of the overall decrease in mutant fish size compared to WT (Fig. 1C).

### Zebrafish *mbnl* mutants have altered movement

**Fig. 2.**
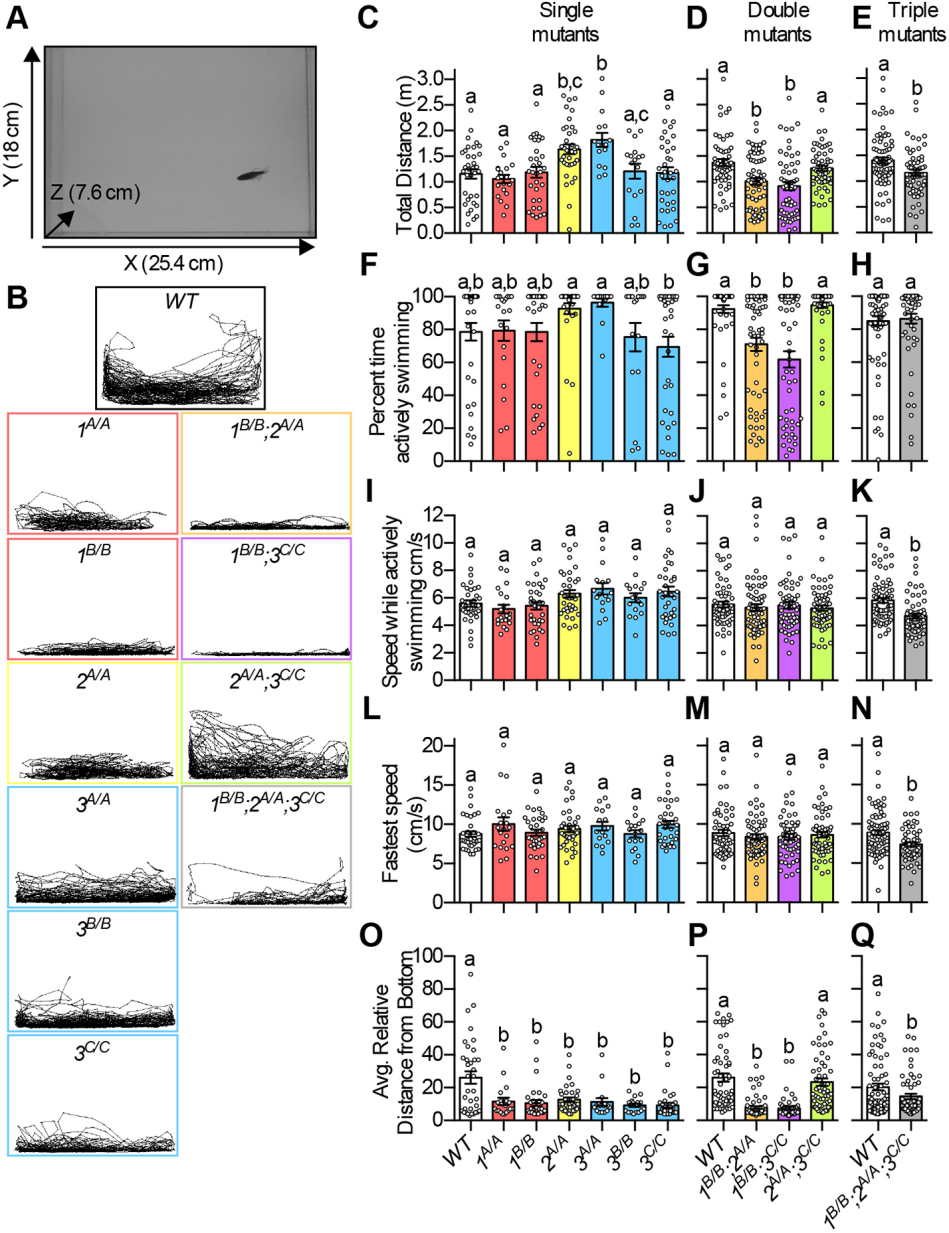
**Zebrafish *mbnl* mutants exhibited altered movement.** (A) Example still image taken from video of swim tests. Software was used to track the center of mass of each fish in the X and Y directions over 5 min. (B) Representative traces of individual fish of each genotype taken during the swim tests. *x*- and *y*-axes are the same as in A. *mbnl1* mutant alleles are denoted as *1^A^* and *1^B^*, *mbnl2* alleles as *2^A^*, and *mbnl3* alleles as *3^A^*, *3^B^* and *3^C^*. (C-E) Total distance that adult WT and single (C), double (D) and triple (E) homozygous *mbnl* mutant fish swam during a 5-min swim test. (F-H) Percentage time actively swimming during the 5-min swim test for single (F), double (G) and triple (H) homozygous *mbnl* mutant fish. Pauses in swimming were removed from the data set, defined as at least 20 consecutive frames (2 s total) with no speed measurement greater than 1.5 cm/s. (I-K) Speed in cm/s during periods of active swimming during the 5-min swim test for single (I), double (J) and triple (K) homozygous *mbnl* mutant fish. (L-N) Fastest speed in cm/s, defined as the fastest 100 consecutive frames (10 s total) during the 5-min swim test, for single (L), double (M) and triple (N) homozygous *mbnl* mutant fish. (O-Q) Average relative distance from the bottom of the tank of fish during the 5-min swim test for single (O), double (P) and triple (Q) homozygous *mbnl* mutant fish. Zero represents the bottom of the tank and 100 represents the top of the tank. In C-Q, each dot represents one fish and data are presented as mean±s.e.m. In C,D,F,G,I,J,L,M,O,P, data were analyzed by ordinary one-way ANOVA with Tukey's multiple comparisons test; in E,H,K,N,Q, data were analyzed by an unpaired Student's *t*-test. Data bars that do not share the same letter above them are significantly different from one another. Raw data and statistical analysis details are in Table S5.

Given that DM impairs motor function, we examined whether zebrafish *mbnl* mutants exhibited altered swimming behavior by introducing individual adult fish to a novel tank and tracking their position over 5 min (Fig. 2A,B). Whereas all single homozygous mutants swam equal or greater distances than WT, two of the double homozygous *mbnl* mutants (*1^B/B^;2^A/A^* and *1^B/B^;3^C/^*^C^) and the *1^B/B^;2^A/A^;3^C/C^* mutants swam significantly decreased distances compared to WT (Fig. 2C-E). In *1^B/B^;2^A/A^* and *1^B/B^;3^C/C^* fish, this phenotype could be explained by a decrease in time spent moving (Fig. 2F-H), as their overall speed during periods of active swimming was not significantly decreased compared to WT, nor was their fastest swimming speed during the recording period (Fig. 2I-N). The *1^B/B^;2^A/A^;3^C/C^* fish, on the other hand, swam significantly more slowly than WT during periods of active swimming, and their fastest swimming speed was less than that of WT (Fig. 2K,N). We suggest that the decreased swim distance in *1^B/B^;2^A/A^* and *1^B/B^;3^C/C^* fish may be primarily behavioral in nature, whereas in *1^B/B^;2^A/A^;3^C/C^* fish it may be due to impaired swimming ability, perhaps as a result of subtle changes in muscle structure and function.

We also observed a striking difference in the positions of *mbnl* mutants and WT fish within the tank, which was quantified by measuring the distance from the bottom of the tank in each frame for each fish and averaging this over the recording period. Except for *2^A/A^;3^C/C^* mutants, all *mbnl* mutants spent significantly more time toward the bottom of the tank than WT fish (Fig. 2O-Q). Overall, these results indicated that loss of *mbnl* function resulted in altered movement in fish, but the exact mechanisms still need to be explored.

### Zebrafish *mbnl* mutants display changes in alternative splicing across tissues

Given the DM-relevant size and movement phenotypes of *mbnl* mutant fish, we asked whether they also exhibited DM-associated alternative splicing changes. The model system used in our initial studies was the extensively characterized suppression of *mbnl1* and *mbnl2* exon 5 inclusion by Mbnl proteins, which is known to affect the subcellular localization and splicing regulatory activity of the encoded proteins (Gates et al., 2011; Terenzi and Ladd, 2010; Tran et al., 2011). The intronic sequences immediately upstream of *mbnl1* and *mbnl2* exon 5, which contain putative YGCY Mbnl protein-binding sites, are highly conserved between human and zebrafish, suggesting functional importance (Figs S4 and S5).

**Fig. 3.**
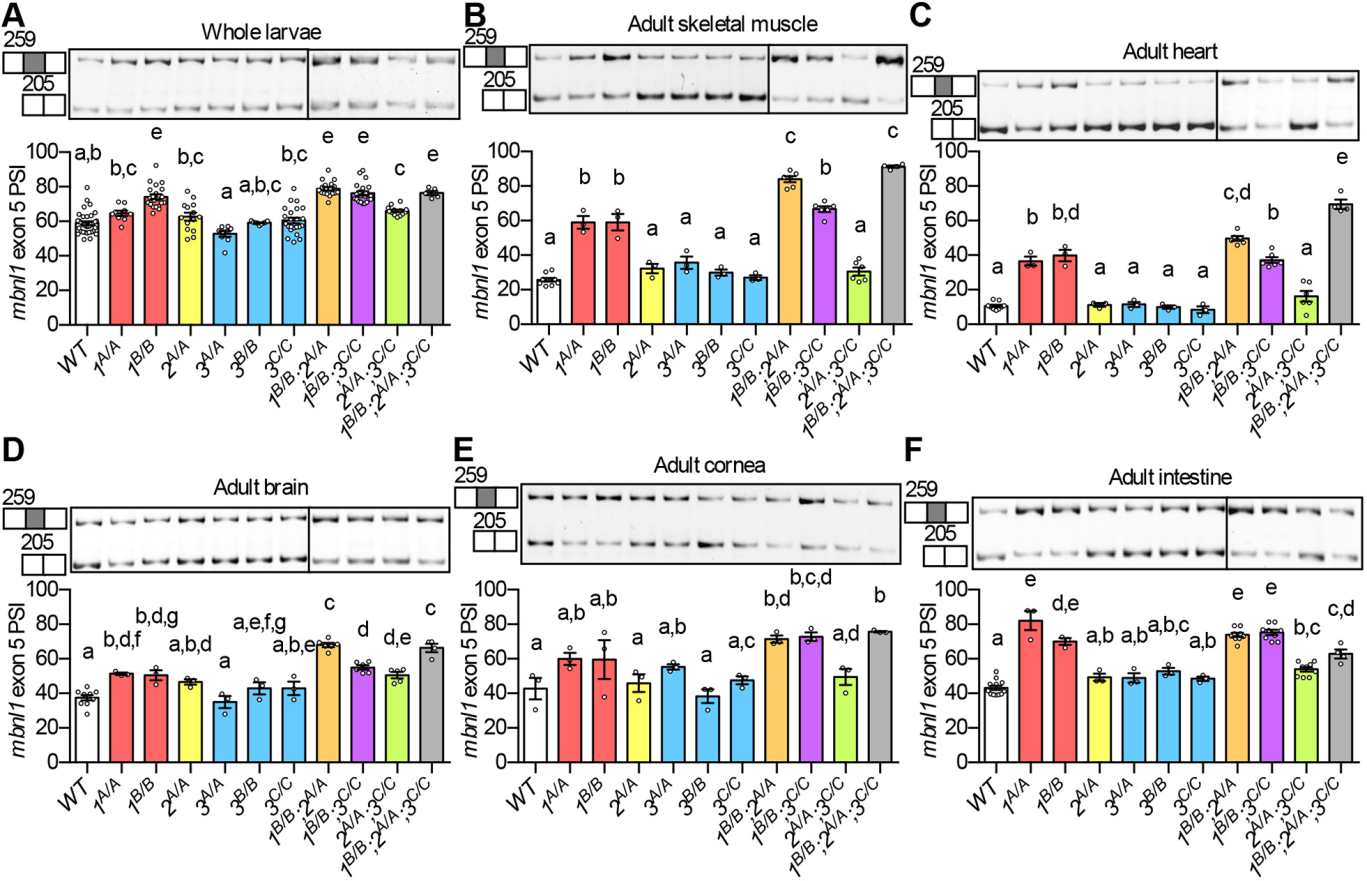
**Alternative splicing of *mbnl1* exon 5 was misregulated across tissues in zebrafish *mbnl* mutants.** (A-F) RT-PCR analysis showing percentage spliced in (PSI) of *mbnl1* exon 5 in WT and *mbnl* mutant whole 5 dpf larvae (A) and in adult skeletal muscle (B), heart (C), brain (D), cornea (E) and intestine (F). *mbnl1* mutant alleles are denoted as *1^A^* and *1^B^*, *mbnl2* alleles as *2^A^*, and *mbnl3* alleles as *3^A^*, *3^B^* and *3^C^*. Representative RT-PCR gels are shown above each graph with band sizes in bp on the left. White boxes represent constitutive exons and gray boxes represent alternative exons. Dividing lines indicate samples run on separate gels. *MBNL1* exon 5 inclusion is increased in human DM1 patients and other DM models (Gates et al., 2011; Terenzi and Ladd, 2010; Tran et al., 2011). In A-F, each dot represents RNA from one adult fish or a pool of five larval fish. Data are presented as mean±s.e.m. Data were analyzed by ordinary one-way ANOVA with Tukey's multiple comparisons test. Data bars that do not share the same letter above them are significantly different from one another. Raw data and statistical analysis details are in Table S5.

We harvested total RNA from whole larval zebrafish and adult skeletal muscle, heart, brain, cornea and intestine. Quantitative RT-PCR (RT-qPCR) analyses of WT fish indicated that *mbnl1*, *mbnl2* and *mbnl3* mRNAs were present in larvae and across adult tissues, and that levels of all three were highest in skeletal muscle and brain (Fig. S1D-F). Inclusion of *mbnl1* exon 5 and *mbnl2* exon 5, as measured by RT-PCR, was significantly elevated in zebrafish *mbnl* mutants across all tissues (Fig. 3; Fig. S6). The phenotype was most dramatic in skeletal muscle and heart, where *mbnl1* and *mbnl2* exon 5 were almost entirely skipped in WT and predominantly included in *1^B/B^;2^A/A^* and *1^B/B^;2^A/A^;3^C/C^* mutants (Fig. 3B,C; Fig. S6B,C). The brain and cornea exhibited *mbnl* splicing patterns similar to one another, but with lower magnitude changes than those observed in muscle (Fig. 3D,E; Fig. S6D,E). Modest but significant splicing changes were observed in whole larvae and intestine (Fig. 3A,F; Fig. S6A,F).

In most tissues, double and triple mutants exhibited larger *mbnl1* and *mbnl2* splicing changes than single mutants (Fig. 3; Fig. S6). This is consistent with the idea that zebrafish Mbnl proteins, like mouse Mbnl proteins, have partially redundant functions when it comes to splicing regulation (Lee et al., 2013; Thomas et al., 2017). Strikingly, the genotypes with the strongest splicing phenotypes, *1^B/B^;2^A/A^* and *1^B/B^;2^A/A^;3^C/C^*, also had dramatic size and movement phenotypes (Figs 1-3; Fig. S6).

In mouse model and DM patient tissues, some regulated exons were much more sensitive than others to changes in overall levels of free MBNL proteins (Wagner et al., 2016). Zebrafish also followed this pattern. For example, in skeletal muscle and heart, *mbnl1* mutation alone was sufficient to increase *mbnl1* exon 5 inclusion, whereas mutation of both *mbnl1* and *mbnl2* was required to increase *mbnl2* exon 5 inclusion (Fig. 3B,C; Fig. S6B,C). Overall, these results suggest that, as in other systems, zebrafish Mbnl proteins work in concert to regulate alternative splicing across tissues.

### Misregulation of alternative splicing is widespread in zebrafish *mbnl* mutants

**Fig. 4.**
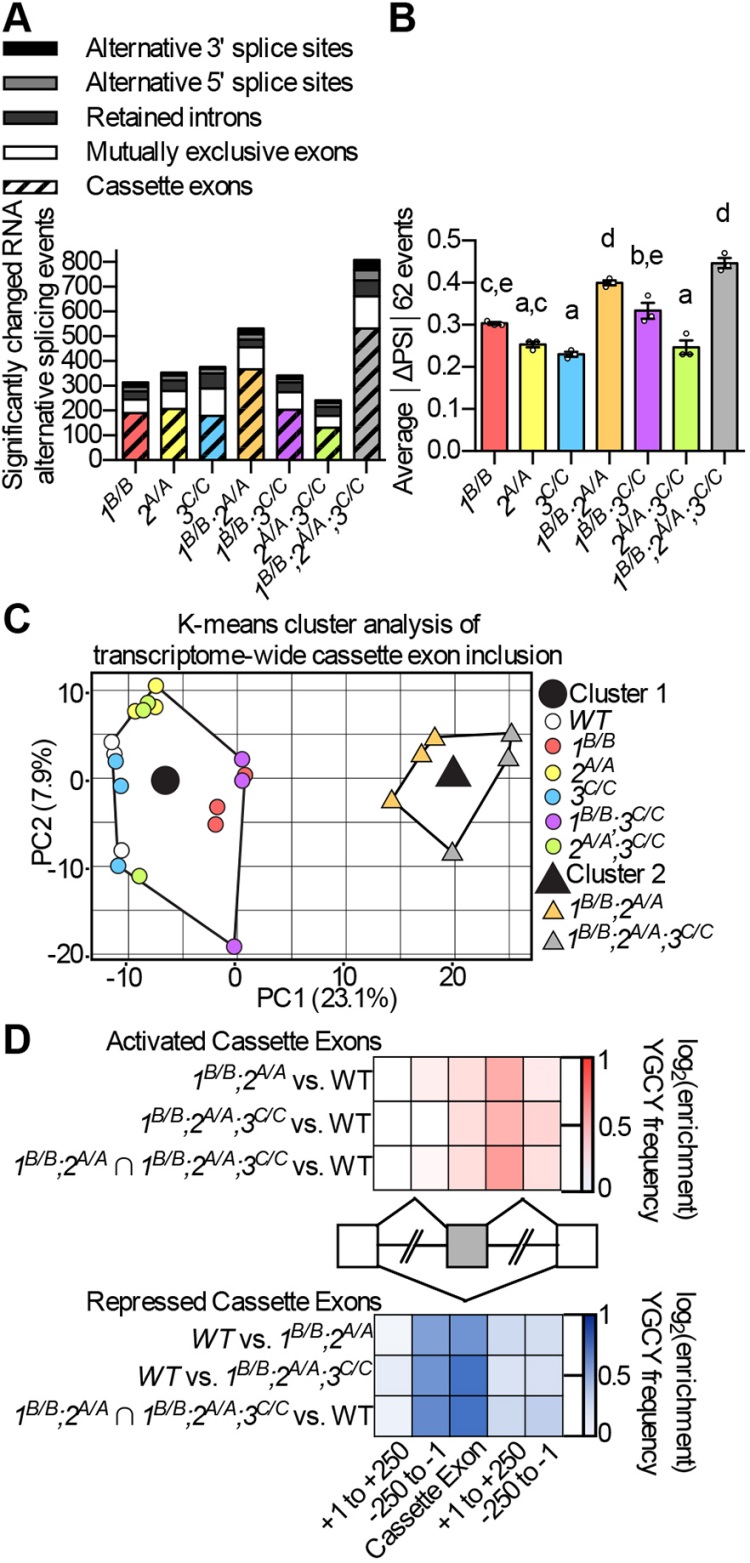
**Zebrafish *mbnl* mutations led to widespread changes in adult skeletal muscle RNA alternative splicing.** (A) Total number of RNA alternative splicing events of different types that were significantly misregulated between WT and *mbnl* mutant adult skeletal muscle, as identified by RNA-Seq. (B) The average absolute value of the change in percentage spliced in (ΔPSI) is shown for the set of 62 cassette exons for which inclusion was significantly misregulated in RNA-Seq analysis of the skeletal muscle of at least four of seven *mbnl* mutant fish lines compared to WT. (C) K-means cluster analysis based on changes in cassette exon inclusion showing that *1^B/B^;2^A/A^* and *1^B/B^;2^A/A^;3^C/C^* mutants cluster closely with each other, while other mutants cluster more closely with WT. Each small circle or triangle represents an individual fish, and the large circle and triangle represent the centers of the clusters. (D) Heat maps showing the enrichment of the previously identified Mbnl protein-binding sequence YGCY (where Y is a pyrimidine) within significantly misregulated cassette exons, in the intronic sequences 250 bp immediately upstream and downstream of those exons, and in the 250 bp of intronic sequences immediately adjacent to the flanking constitutive exons. Activated cassette exons are those in which Mbnl proteins regulated inclusion positively, and repressed cassette exons are those for which inclusion is decreased by Mbnl proteins. The YGCY enrichment analysis was performed for the set of cassette exons that were significantly misregulated in *1^B/B^;2^A/A^* fish, in *1^B/B^;2^A/A^;3^C/C^* fish, and for the set of misregulated exons that overlapped between the two (*1^B/B^;2^A/A^*∩*1^B/B^;2^A/A^;3^C/C^*). In A-D, the *mbnl1* mutant allele is denoted as*1^B^*, the *mbnl2* allele as *2^A^* and the *mbnl3* allele as *3^C^*. In B, each dot represents one fish. Data are presented as mean±s.e.m. Data were analyzed by ordinary one-way ANOVA with Tukey's multiple comparisons test. Data bars that do not share the same letter above them are significantly different from one another. Raw data and statistical analysis details are in Table S5.

To understand the genome-wide impact of *mbnl* mutation on alternative splicing, we performed RNA-Seq analysis using RNA isolated from the skeletal muscle of adult WT, *1^B/B^*, *2^A/A^*, *3^C/C^*, *1^B/B^;2^A/A^*, *1^B/B^;3^C/C^*, *2^A/A^;3^C/C^* and *1^B/B^;2^A/A^;3^C/C^* zebrafish. There were no significant changes in the normalized counts of *mbnl1* and *mbnl3* mRNAs in mutants compared to WT, while *mbnl2* mRNA levels were slightly decreased when *mbnl2* was mutated (Fig. S1H-J). Hundreds of significantly misregulated RNA alternative splicing events were detected in all *mbnl* mutants, with misregulated cassette exons outnumbering alternative 3′ and 5′ splice sites, retained introns and mutually exclusive exons (Fig. 4A; Table S4). The *1^B/B^;2^A/A^* and *1^B/B^;2^A/A^;3^C/C^* fish had the largest number of significantly misregulated splicing events (Fig. 4A). Surprisingly, *2^A/A^;3^C/C^* mutants exhibited fewer overall dysregulated alternative splicing events than *2^A/A^* mutants or *3^C/C^* mutants (Fig. 4A).

We also analyzed the magnitude of the splicing change [absolute value of the change in percentage spliced in (ΔPSI) between WT and mutant] for the 62 cassette exon events that were significantly misregulated in the majority of *mbnl* mutants (at least four of seven). The highest magnitude of splicing change across events was also observed in *1^B/B^;2^A/A^* and *1^B/B^;2^A/A^;3^C/C^* fish (Fig. 4B). These results suggest that zebrafish Mbnl proteins, like mouse orthologs, have at least partially redundant functions (Lee et al., 2013; Thomas et al., 2017).

We next asked which genotypes were most similar in their overall splicing phenotypes. All cassette exon splicing changes that were observed in any mutant compared to WT were compiled into a single list, and the top 800 events with the largest variability in PSI were identified. Using the gap statistic, we determined that all samples could be clustered into two groups based on the splicing phenotype for those events. A K-means cluster analysis with two centers was then performed, in which all WT and mutant samples were analyzed for their similarity in PSI for the 800 events. The analysis indicated that all *1^B/B^;2^A/A^* and *1^B/B^;2^A/A^;3^C/C^* fish were more similar to each other than they were to WT fish or any of the other *mbnl* mutant fish (Fig. 4C). Taken together, these results indicated that compound loss of function of zebrafish Mbnl proteins led to widespread and robust changes in alternative splicing.

We analyzed the frequency of the Mbnl protein binding motif, YGCY, within and surrounding the cassette exons that were significantly misregulated in *1^B/B^;2^A/A^* and *1^B/B^;2^A/A^;3^C/C^* mutants as well as those misregulated cassette exons that were in common between the two mutants. YGCY motifs were enriched in the introns downstream of exons for which inclusion was activated by the presence of Mbnl proteins, while YGCY motifs were enriched upstream and within the cassette exons that were repressed by Mbnl proteins (Fig. 4D). This was consistent with findings in other DM model systems, and suggests that zebrafish Mbnl proteins played a direct role in regulating the inclusion of cassette exons (Du et al., 2010; Goers et al., 2010; Oddo et al., 2016; Wang et al., 2012).

### Zebrafish *mbnl* mutants exhibit disease-relevant alternative splicing changes

**Fig. 5.**
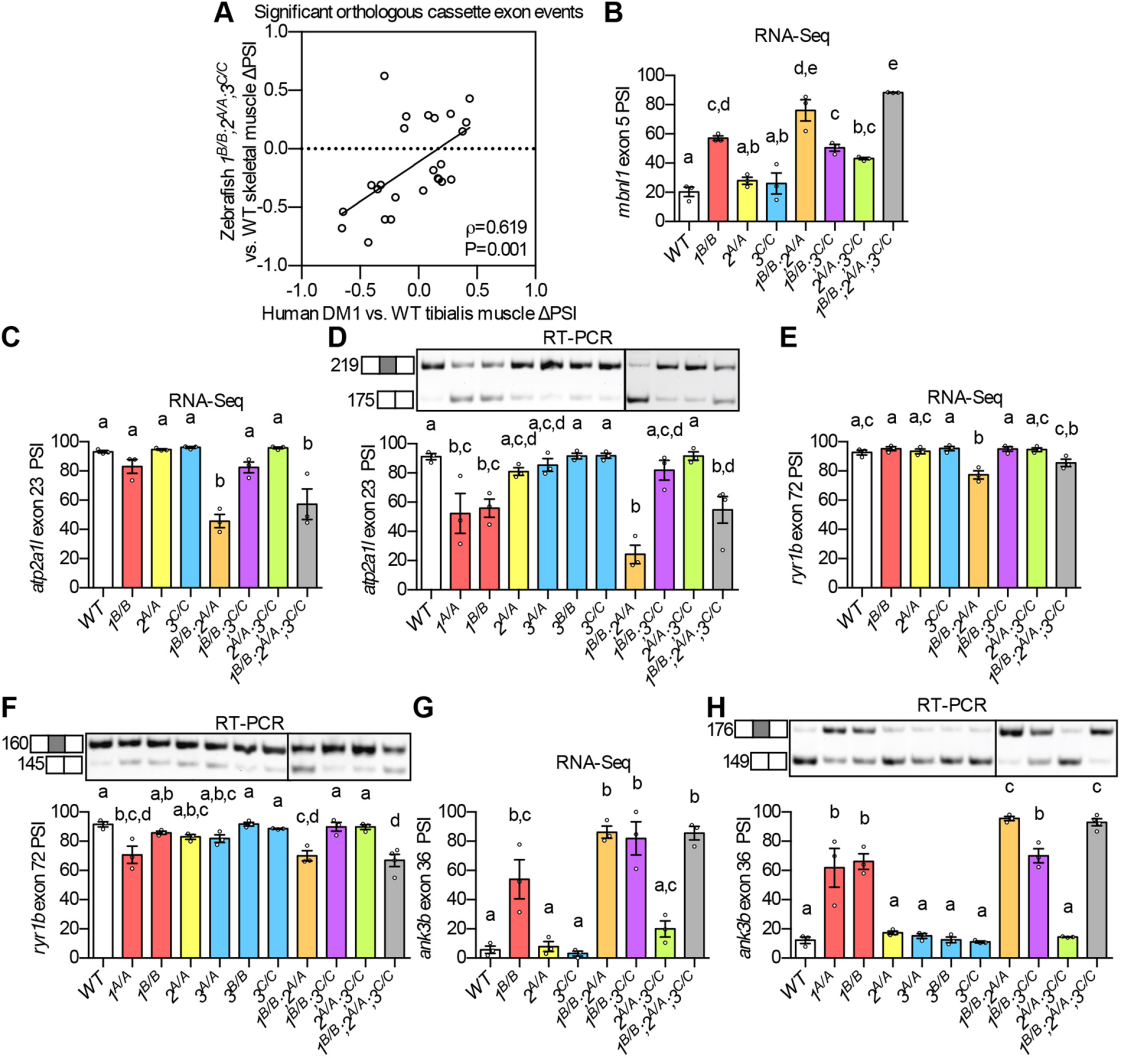
**Misregulation of many DM-associated alternative splicing events was conserved in *mbnl* mutants.** (A) ΔPSI between mutant and WT are shown for orthologous exons in zebrafish *1^B/B^;2^A/A^;3^C/C^* skeletal muscle and in tibialis muscle from human DM1 patients. (B-H) RNA-Seq (B,C,E,G) and RT-PCR (D,F,H) analyses showing PSI of *mbnl1* exon 5 (B), *atp2a1l* exon 23 (C,D), *ryr1b* exon 72 (E,F), and *ank3b* exon 36 (G,H) in WT and *mbnl* mutant adult zebrafish skeletal muscle. Orthologous human exon inclusion in DM patients is increased for *MBNL1* and *ANK3*, and decreased for *ATP2A1* and *RYR1* (Freyermuth et al., 2016; Gates et al., 2011; Kimura et al., 2005; Terenzi and Ladd, 2010; Tran et al., 2011; Zhao et al., 2015). In A-H, *mbnl1* mutant alleles are denoted as *1^A^* and *1^B^*, *mbnl2* alleles as *2^A^*, and *mbnl3* alleles as *3^A^*, *3^B^* and *3^C^*. In A, ρ is the Spearman's rank correlation coefficient. In B-H, data are presented as mean±s.e.m. Each dot represents RNA from one fish. Representative gels are shown above each RT-PCR graph with band sizes in bp shown on the left. White boxes represent constitutive exons and gray boxes represent alternative exons. Dividing lines indicate samples run on separate gels. Data were analyzed by ordinary one-way ANOVA with Tukey's multiple comparisons test. Data bars that do not share the same letter above them are significantly different from one another. Raw data and statistical analysis details are in Table S5.

Given the widespread changes in alternative splicing in *mbnl* mutant zebrafish, we asked whether these changes were conserved with those identified in human DM patients. Using publicly available datasets, we identified cassette exons for which inclusion was significantly misregulated in DM1 tibialis anterior muscle biopsy tissues (Wang et al., 2019) or in DM1 patient-derived post-mitotic myotubes (Reddy et al., 2019) compared to healthy control tissues, and compared them with the zebrafish RNA-Seq data. We identified 25 orthologous cassette exons that were misregulated in both *1^B/B^;2^A/A^;3^C/C^* mutant fish and in DM1 tibialis muscle, and 40 that were misregulated in both *1^B/B^;2^A/A^;3^C/C^* mutant fish and in the DM1 myotubes (Fig. 5A; Fig. S7A and Table S5). The inclusion of these alternative exons tended to be misregulated in the same direction in zebrafish DM models and in DM1 patient-derived tissues (Fig. 5A; Fig. S7A).

As anticipated, *mbnl1* exon 5 and *mbnl2* exon 5 appeared on both lists of orthologous misregulated cassette exons, and PSI values were strikingly similar as determined by RNA-Seq and RT-PCR (Figs. 3B and 5B; Figs. S6B and S7B). Two other randomly selected orthologous exons, *aplp2* exon 7 and *atp6v1h* exon6, also showed similar splicing phenotypes by RNA-Seq and RT-PCR, and contained potential YGCY Mbnl-binding motifs in their surrounding intronic sequences (Fig. S7C-F, Figs S8 and S9). Fig. 5A and Fig. S7A do not represent an exhaustive list of orthologous misregulated exons, as a manual literature search revealed several additional orthologs of disease-associated exons that were misregulated in *mbnl* mutant model fish, some of which were only annotated in an earlier zebrafish genome assembly (GRCz10, Table S4). For example, the decreased inclusion of alternative exons within the human *ATP2A1* and *RYR1* genes contributes to altered calcium homeostasis in DM muscle, and we detected decreased inclusion of orthologous exons in zebrafish *mbnl* mutants (Fig. 5C-F; Figs S10 and S11) (Kimura et al., 2005; Zhao et al., 2015). Likewise, we observed dramatic mis-splicing of zebrafish *ank3b* exon 36, the human ortholog of which is known to be misregulated in human DM1 heart samples (Fig. 5G,H; Fig. S12) (Freyermuth et al., 2016).

We also analyzed gene expression level differences in skeletal muscle, focusing on *1^B/B^;2^A/A^;3^C/C^* fish, which had the most severe alternative splicing and physical phenotypes. Over a thousand genes had significantly changed expression levels in the triple homozygous *mbnl* mutants compared to WT, suggesting significant molecular dysfunction in muscle (Table S6). We then compared the gene ontology (GO) classifications of dysregulated genes in the *1^B/B^;2^A/A^;3^C/C^* mutant zebrafish skeletal muscle to those uncovered in our analysis of published RNA-Seq data from human DM1 myotubes (Reddy et al., 2019). We found that many functional pathways related to muscle development were dysregulated in both *1^B/B^;2^A/A^;3^C/C^* zebrafish and human DM1 myotubes (Fig. S13 and Table S6). Taken together, these results indicate that the overall coordinated splicing regulation program of the Mbnl proteins, as well as gene expression changes, were well conserved between zebrafish and humans.

## DISCUSSION

For the first time, we have modeled DM-associated *MBNL* loss of function by generating a complete panel of vertebrate *mbnl* single, double and triple homozygous mutants. These fish exhibited molecular and physical phenotypes similar to those observed in humans with DM and mouse models, including decreased body size, impaired movement and widespread changes in alternative splicing. We propose that zebrafish *1^B/B^;2^A/A^* and *1^B/B^;2^A/A^;3^F/F^* mutants, which exhibited the most dramatic phenotypes, represent severe forms of DM1, with long CTG repeats in which most, but probably not all, MBNL proteins are sequestered, while other double and single *mbnl* mutants model more moderate forms of the disease, in which MBNL sequestration is less robust.

We were initially surprised that homozygous *mbnl2* mutant zebrafish were viable to adulthood, as this contrasted with the dramatic morphological phenotypes of *mbnl2* morpholino-injected larvae (Machuca-Tzili et al., 2011). We propose that partially retained protein function due to the presence of one remaining zinc finger pair (Fig. 1B) or morpholino off-target effects may explain the discrepancy in phenotype between *mbnl2* morphants and mutants. We favor the latter explanation, as Mbnl2 function was not required for survival in mice (Charizanis et al., 2012). We are confident that *2^A/A^* mutants represented at least a partial loss of function given their splicing phenotypes and reduced *mbnl2* mRNA levels (Fig. 4; Fig. S1B), but definitive null alleles would be needed to determine whether *mbnl2* is required for viability in zebrafish.

The viability of zebrafish *1^B/B^;2^A/A^* and *1^B/B^;2^A/A^;3^C/C^* mutants was also surprising, as this differed from previous findings in mouse models (Lee et al., 2013; Thomas et al., 2017). Most muscle-specific double and triple *Mbnl* mutant mice died during the neonatal period when they transitioned abruptly to breathing, but the small fraction that made it through this transition survived to adulthood (Thomas et al., 2017). Perhaps *1^B/B^;2^A/A^* and *1^B/B^;2^A/A^;3^C/C^* fish were better able to survive because larval fish underwent a gradual transition between receiving oxygen through diffusion and through the gills. In addition, unlike the more tissue-restricted expression of mouse *Mbnl* genes (Charizanis et al., 2012; Lee et al., 2013; Poulos et al., 2013), all three zebrafish *mbnl* genes were expressed broadly (Fig. S1D-F), and maybe the presence of partially functional Mbnl2 protein across tissues including skeletal muscle was sufficient for zebrafish survival. Although viable, the triple mutants had more severe physical impairments than the single or double mutants. They did not mate spontaneously, required supplemental feeding, and all died by 18 months of age.

Regardless of whether *mbnl* mutant fish are nulls or hypomorphs, they are valid models of DM, as sequestration of MBNL proteins by CUG/CCUG repeats is likely not complete in the human disease state. The robust molecular and physical phenotypes, yet viability, of *1^B/B^;2^A/A^* and *1^B/B^;2^A/A^;3^C/C^* fish will make them particularly useful tools for studying how loss of *mbnl* function contributes to disease-relevant phenotypes. The compound *Mbnl* mutant mouse models that most closely mimic DM phenotypes are challenging to breed in large numbers and most die during the neonatal period, making studies of adult phenotypes difficult (Thomas et al., 2017). In contrast, hundreds of *1^B/B^;2^A/A^* and *1^B/B^;2^A/A^;3^C/C^* zebrafish can be generated in a single cross or through *in vitro* fertilization, providing the statistical power to analyze subtle or variable phenotypes, test potential therapeutics, or screen for genetic modifiers of *mbnl* activity in both development and adulthood.

Zebrafish *mbnl* mutants had physical phenotypes that are relevant to human DM disease and mouse DM models. Adult *1^B/B^;2^A/A^*, *1^B/B^;3^C/C^* and *1^B/B^;2^A/A^;3^C/C^* fish were significantly smaller than their WT tank mates (Fig. 1C). Neonatal muscle-specific compound mutant mice likewise were substantially smaller than their littermates (Thomas et al., 2017). In the case of fish, the size phenotype did not arise early in development, as 7 dpf larval *mbnl* mutant fish were not smaller than WT fish (Fig. 1D,E; Fig. S2D). We do not know the mechanism underlying the size phenotype in *mbnl* mutant fish, but one possibility is DM-relevant impaired movement leading to decreased ability to access food. Consistent with this idea, we found that *1^B/B^;2^A/A^*, *1^B/B^;3^C/C^* and *1^B/B^;2^A/A^;3^C/C^* fish swam a significantly decreased distance compared to WT fish during a 5-min swim test (Fig. 2D,E). Swimming ability appeared to be most impaired in the triple mutant fish, which exhibited both decreased active swimming speed and decreased fastest swimming speed compared to WT (Fig. 2K,N). Given the widespread dysregulation of both alternative splicing and gene expression that was present in *mbnl* mutant skeletal muscle, it seems likely that muscle function is perturbed, which could contribute to the observed impaired movement (Fig. 4A; Fig. S13 and Table S6). We observed a subtle decrease in the absolute cross-sectional area of individual muscle fibers in *1^B/B^;2^A/A^* and *1^B/B^;2^A/A^;3^C/C^* fish compared to WT. However, it is difficult to determine whether this is an indication of muscle pathology, or is merely due to decreased body size, which is roughly associated with decreased muscle fiber size, but not through a simple linear relationship (Jimenez et al., 2013).

We also found that almost all genotypes of *mbnl* mutant fish stayed closer to the bottom of the tank on average than WT fish (Fig. 2O-Q). Lower tank position is a well-characterized indicator of anxiety in zebrafish (Cachat et al., 2010), and it is possible that the position phenotype in the *mbnl* mutants was due to increased anxiety. We think that a more likely explanation is that the *mbnl* mutant fish stayed at the bottom of the tank due to motor dysfunction or a morphological defect. Our future studies will explore in depth the mechanisms underlying decreased size and altered swimming behavior in *mbnl* mutant fish.

Like humans with DM and mouse models, zebrafish *mbnl* mutants exhibited widespread changes in alternative splicing (Fig. 4). Splicing changes in zebrafish *mbnl* mutants occurred in both larvae and adults, and across many DM-relevant tissues, including skeletal muscle, heart, brain and intestine (Fig. 3; Fig. S6). Mis-splicing was also observed in the cornea, suggesting that zebrafish *mbnl* mutants may also model the genetic disorder Fuchs endothelial corneal dystrophy, a subtype of which was recently shown to be caused by an expanded CUG repeat that is expressed from an intron of the *TCF4* gene and is associated with MBNL protein sequestration and mis-splicing (Fig. 3E; Fig. S6E) (Mootha et al., 2017, 2015; Wieben et al., 2017; Winkler et al., 2018). Hundreds of alternative splicing changes were observed when each of the zebrafish *mbnl* genes was mutated alone, suggesting that each plays a role in splicing regulation (Fig. 4A). Consistent with mouse studies was the finding that splicing changes were more numerous and larger in magnitude in double and triple homozygous zebrafish mutants than in single homozygous mutants, indicating partially redundant functions of Mbnl proteins (Fig. 4A,B) (Lee et al., 2013; Thomas et al., 2017). Notable exceptions were the *2^A/A^;3^C/C^* mutants, which exhibited fewer overall dysregulated alternative splicing events and lacked the swimming position phenotype of *2^A/A^* mutants and *3^C/C^* mutants (Figs. 2P and 4A). In mouse models, many cassette exon events were shown to be dysregulated in opposite directions in *Mbnl3* mutants and other *Mbnl* mutants (Thomas et al., 2017). Perhaps opposing splicing regulation could be a contributing factor to the mild phenotypes of *2^A/A^;3^C/C^* mutants.

Our data indicated that, just like other model organisms, zebrafish Mbnl*-*mediated alternative splicing regulation was quite complex. Mbnl proteins suppressed inclusion of some exons, while they promoted inclusion of others (Fig. 5A; Fig. S7A). As observed in other organisms, YGCY putative Mbnl-binding motifs were enriched upstream of exons that were suppressed by zebrafish Mbnl proteins, and enriched downstream of exons for which inclusion was promoted (Fig. 4D) (Du et al., 2010; Goers et al., 2010; Oddo et al., 2016; Wang et al., 2012). Also similar to observations in other systems, some zebrafish exons were more sensitive to changes in overall *mbnl* concentration than others (Wagner et al., 2016). For example, in skeletal muscle, *mbnl2* exon 5 required the loss of both *mbnl1* and *mbnl2* function for increased inclusion, whereas *mbnl1* exon 5 only required the loss of *mbnl1* function (Figs. 3B and 5B; Figs. S6B and S7B).

Many of the alternative splicing changes observed in *mbnl* mutant DM model fish were conserved with changes observed in DM1 patient tissues (Fig. 5; Fig. S7 and Table S5). These included several that are known to contribute to disease phenotypes, such as changes in *ATP2A1* and *RYR1* splicing that contribute to dysregulation of calcium homeostasis (Kimura et al., 2005; Zhao et al., 2015). However, not all mis-splicing events were conserved between species. For example, *mbnl* mutants did not exhibit changes in alternative splicing of the zebrafish orthologs of *CLCN1*, the splicing misregulation of which contributes to myotonia in DM, so the zebrafish may not be an appropriate model for studying myotonia, although it can be used to study many other important disease phenotypes (Lueck et al., 2007a,b).

These new zebrafish models, and the accompanying RNA-Seq data that we generated, will be valuable in future studies of how individual Mbnl proteins and specific alternative splicing and gene expression changes contribute to DM-relevant phenotypes. For example, exons that were misregulated in all zebrafish *mbnl* mutants except for *2^A/A^;3^C/C^* mutants are strong candidates for contributing to the tank position phenotype (Fig. 2O-Q). Likewise, exons for which mis-regulation was not conserved between humans with DM and zebrafish are candidates for contributing to disease-related changes in muscle histology, such as central nuclei, that were not observed in the zebrafish *mbnl* mutants (Fig. S3). Zebrafish DM models complement other existing vertebrate model systems because hundreds of larval or adult fish can be generated easily to study subtle or variable phenotypes and to test potential therapeutics. Additionally, transparent larval zebrafish can be used to directly study disease-related phenotypes, such as altered gut motility and heart abnormalities, in live animals.

## MATERIALS AND METHODS

### Generation of mutant zebrafish

All zebrafish (*Danio rerio*) experiments were performed with the guidance and approval of the University of Oregon Institutional Animal Care and Use Committee (PHS assurance number D16-00004, protocols AUP-15-98 and AUP-20-16). Guide RNAs (gRNAs) targeting zebrafish (*Danio rerio*) *mbnl1*, *mbnl2* and *mbnl3* were designed using the Chop Chop website (http://chopchop.cbu.uib.no). DNA templates for the gRNAs were generated by a template-free Phusion polymerase (New England Biolabs) PCR reaction using a common scaffold primer (gRNA scaffold, Table S1) and a gene-specific primer (*mbnl1* gRNA1, *mbnl1* gRNA2, *mbnl2* gRNA, *mbnl3* gRNA1 or *mbnl3* gRNA2, Table S1), then cleaned using the QIAquick PCR Purification Kit (Qiagen). gRNAs were transcribed from DNA templates using a MEGAscript kit (Ambion) and purified by phenol-chloroform extraction and isopropanol precipitation. Cas9 RNA was generated by linearizing the pT3TS-nls-zCas9-nls plasmid (Jao et al., 2013) with XbaI, purifying it using the QIAquick Gel Extraction Kit (Qiagen), performing an *in vitro* transcription reaction using the T3 mMESSAGE kit (Invitrogen), and purifying the RNA using the RNeasy Mini kit (Qiagen). AB zebrafish embryos were microinjected at the one-cell stage with 1-2 nl of a mixture containing 100 ng/µl Cas9 mRNA, 50 ng/µl gRNA and Phenol Red, and raised to adulthood. Mosaic mutants were identified by PCR amplification and Sanger sequencing of fin clip DNA using primers specific to the targeted region (Tables S1 and S2), and outcrossed to WT AB zebrafish to generate heterozygotes. Fish with predicted loss-of-function mutations were identified by Sanger sequencing (Table S2), and further crossed to generate single, double and triple *mbnl1*, *mbnl2* and *mbnl3* homozygotes. Fish were genotyped by restriction fragment length polymorphism analysis using the primers and restriction enzymes indicated in Table S2. Sperm from all zebrafish mutant lines were cryopreserved and are available upon request from the corresponding author. Roughly equal numbers of males and females were used for all experiments involving adult fish.

### Measurement of zebrafish size

Young adult fish (2-4 months old) (Fig. 1C; Fig. S2A-C) were anesthetized in 168 mg/l tricaine methane sulfonate, photographed against a white background with a ruler using a tablet computer, fin clipped and genotyped. To ensure identical density and feeding conditions, fish were compared with others from the same clutch (Fig. S2A-C) or different clutches that had been raised together in the same tank (Fig. 1C). For measurement of larval fish, unfed embryos of different genotypes were raised to 7 dpf in separate dishes at a density of one fish per ml embryo medium (Fig. 1D; Fig. S2D), with the exception of the larvae in Fig. 1E, which were grown in the same dish and genotyped after measurement. Fish were anesthetized in 168 mg/ml tricaine methane sulfonate, laid out on a microscope slide in 3% methylcellulose, and imaged using a Leica M165FC microscope. An investigator that was blinded to genotype measured the distance from snout to caudal peduncle or posterior tip of the notochord (standard length) of each fish (Parichy et al., 2009). Data were analyzed by ordinary one-way ANOVA with multiple comparison correction (Table S5).

### Alternative splicing analysis by RT-PCR

Fish were euthanized by tricaine methane sulfonate overdose (larvae) or hypothermic shock (adults). Tissues of interest were dissected and flash frozen in 1 ml Trizol (Ambion), thawed, and homogenized with a mortar and pestle (larvae) or Bullet Blender Storm 24 (adult tissues). Chloroform (200 µl) was added to each tube followed by mixing, centrifugation at 12,000 ***g*** for 10 min at 4°C, transfer of the aqueous phase to a separate tube, addition of 200 µl ethanol and binding of sample to an RNeasy mini kit column (Qiagen). RNA was washed and eluted according to the manufacturer's instructions, and the concentration was measured using a NanoDrop 2000 (Thermo Fisher Scientific).

RNA (20-200 ng) was reverse transcribed with Superscript II Reverse Transcriptase (Invitrogen) according to the manufacturer's instructions using gene-specific reverse (R) primers located in the exon downstream of the regulated exon of interest (Table S1). The resulting complementary DNA (cDNA) was amplified by 28-31 cycles of PCR using Taq polymerase and the forward (F) and reverse (R) primers indicated in Table S1. Samples were separated by electrophoresis on a 6% *bis*-Acrylamide (19:1) gel that was stained overnight with 1× SYBR Green I Nucleic Acid Gel Stain (Invitrogen). The gel was imaged and quantified using an AlphaImagerHP (Alpha Innotech). The background-corrected sum of each band was measured and the percentage exon inclusion was calculated using the following formula: [(exon included sum)/(exon included sum+exon excluded sum)]×100. Data were analyzed by ordinary one-way ANOVA with multiple comparison correction.

### RNA-Seq analysis

RNA was extracted using the RiboPure RNA Purification Kit (Invitrogen AM1924) from epaxial skeletal muscle from the tails of three biological replicates of adult WT and mutant fish. RNA quality was examined using the Fragment Analyzer RNA Analysis DNF-471 kit (Advanced Analytical) and all RNA quality number (RQN) values were >8.0. Ribosomal RNA was depleted from 200 µg of the RNA using the NEBNext rRNA Depletion Kit (NEB E6310X), and then a cDNA library was prepared using the NEBNext Ultra RNA Library Prep Kit for Illumina (NEB E7530L). The libraries were checked for quality using the Fragment Analyzer NGS Analysis DNF-474 kit (Advanced Analytical) and quantitated using the KAPA Library Quantification Kit (KAPA Code KK4824). The completed libraries were then pooled in equimolar amounts and sequenced on an Illumina Next-Seq 500. A minimum of 60 million paired-end 75×75 reads were obtained for each library.

BCL files were demultiplexed and converted to fastq files using BCL2Fastq (version 2.16.0.10). The fastq files were then checked for quality using FastQC (version 0.11.8) and aligned to the GRCz11 zebrafish genome using STAR (version 2.5.1b). The alternative splicing events were then analyzed using rMATS (version 4.0.2), grouping the biological replicates of each mutant and comparing them against WT. A similar preliminary analysis of the same sequencing data was performed using GRCz10 and rMATS (version 4.0.2). Significant mis-splicing events were categorized as having a false discovery rate (FDR) <0.10. Cluster analysis was performed with R and visualized with the factoextra package (version 1.0.5) using the top 800 most variable cassette exon splicing events in PSI values. Data were first tested using the gap statistic and then K-means clustering was performed with two centers. The data for human DM1 tibialis anterior comparisons were downloaded from NCBI Gene Expression Omnibus (GEO), accession number GSE86356 (Wang et al., 2019). Data for the DM1-derived postmitotic myotubes were downloaded from NCBI Sequence Read Archive (SRA), study SRP158284 (Reddy et al., 2019). The data were processed as described above and then compared to the zebrafish data for orthologous mis-splicing events. Orthologous exons were found using a custom python script and using tblastx to confirm. Exons were counted as orthologous if the genes from which they were transcribed were at least 75% conserved between species, the exon was in the same place in the transcript (equal exon number in at least one annotated transcript), and if the exon had an evalue from tblastx of lower than 0.05. Expression levels of *mbnl* mRNAs were determined using DESeq2. WT unfertilized egg RNA-Seq data were downloaded from NCBI SRA, accession number SRS4049994 (Mehjabin et al., 2019).

For GO analysis, BCL files were demultiplexed and converted to Fastq files using BCL2Fastq (version 2.16.0.10). The Fastq files were then checked for quality using FastQC (version 0.11.8) and aligned to the GRCz10 genome using STAR (version 2.5.1b). Reads counts for WT and *1^B/B^;2^A/A^;3^C/C^* zebrafish, and for WT and DM1 myotubes were obtained using Stringtie (version 1.2.3). Differential expression analysis was performed using DESeq2 (version 1.28.1). Significantly differentially expressed genes (compared to their respective controls) were filtered at an absolute log_2_ value of 1.0 and a FDR value of less than 0.05, and then analyzed using PANTHER Over-representation Test (GO database DOI: 10.5281/zenodo.3873405, released 1 June 2020). A dot plot was created using GGPlot2 (version 3.3.2) showing significantly over-represented GO terms (FDR<0.05) in *1^B/B^;2^A/A^;3^C/C^* zebrafish compared to WT. General GO categories with >100 annotated genes were omitted from the plot, and GO categories that overlapped with those over-represented in DM1 myotubes were highlighted.

Enrichment of putative YGCY Mbnl protein-binding motifs was analyzed for the sets of cassette exons for which inclusion was significantly increased (FDR<0.05, ΔPSI>0.2) or decreased (FDR<0.05, ΔPSI<−0.2) compared to WT in *1^B/B^;2^A/A^* mutants, *1^B/B^;2^A/A^;3^C/C^* mutants or in the intersection of both sets (*1^B/B^;2^A/A^* ∩ *1^B/B^;2^A/A^;3^C/C^*). For each set of cassette exons, the frequency of YGCY motifs (GCTT, CGCT, TGCT and GCGC) was compared to the frequency of control 4-mers with identical A+T and CpG content within the following regions: cassette exons (normalized for analyzed sequence length), intronic sequences 1-250 bp immediately 5′ or 3′ of the cassette exons, intronic sequences 1-250 bp 3′ of the upstream constitutive exons, and intronic sequences 1-250 bp 5′ of the downstream constitutive exons. The log_2_ [(enrichment of YGCY motifs relative to control motifs in each region associated with regulated cassette exons)/(enrichment of YGCY motifs relative to control motifs in each region associated with non-regulated exons)] was plotted in heatmap form.

### Analysis of *mbnl* gene expression

RT-qPCR was used to measure *mbnl* RNA levels in zebrafish tissues. Total RNA was prepared from 5 dpf larvae and adult tissues using the same procedure as for alternative splicing analysis. RNA was treated with TURBO DNase (Ambion) according to the manufacturer's instructions, then reverse transcribed with an oligo(dT)_20_ primer using the Superscript III cDNA First Strand Synthesis kit (Invitrogen). The quantitative PCR reaction was set up using a KAPA SYBR FAST ABI Prism kit (KAPA Biosystems) according to the manufacturer's instructions and run on a Quant Studio 3 System (Thermo Fisher Scientific) using the default settings for SYBR Green reagents and the fast run mode in the QuantStudio Design and Analysis Software v.1.4.2. The comparative C_T_ (ΔΔC_T_) method was used to calculate relative mRNA levels. Five biological samples of each genotype or tissue type were run in triplicate and data were normalized to the expression of the housekeeping gene *eef1a1l1* (Vanhauwaert et al., 2014). Primers used for RT-qPCR are shown in Table S1.

Western blots were performed using 15-50 µg of protein lysates that were prepared in RIPA buffer (Boston Bioproducts) with complete mini EDTA-free protease inhibitor (Roche) from HeLa cells (positive control for MBNL1 and MBNL2 protein expression), whole 5 dpf zebrafish larvae, adult skeletal muscle, heart, brain, intestine and cornea, or purchased from Santa Cruz Biotechnology (human placenta extract used as a positive control for MBNL3 protein expression). The antibodies tested included Santa Cruz Biotechnology anti-MBNL1 (D-4) sc-515374 (1:250), Santa Cruz Biotechnology anti-MBNL1 (4A8) sc-136165 (1:500), Millipore anti-MBNL1 ABE-241 (1:1000), Abcam anti-MBNL2 ab105331 (1:250), Sigma-Aldrich anti-MBNL3 SAB1411751 (1:150) and Sigma-Aldrich anti-Actin (20-33) A5060 (1:1000). Santa Cruz Biotechnology goat anti-rabbit IgG-horseradish peroxidase (HRP) sc-2004 (1:2000) and Sigma-Aldrich goat anti-mouse IgG FC-specific HRP (1:2000) were used as secondary antibodies.

### Zebrafish behavior assays

Age-matched single adult zebrafish were placed into a novel environment consisting of a custom glass aquarium measuring 18 cm deep×25.4 cm long×7.6 cm wide (Fig. 2A). The fish were monitored with a Logitech camera during a 5-min time frame to characterize basic exploratory swimming behavior. The raw tracking data was analyzed with custom software (DaniOPEN, https://github.com/stednitzs/daniopen) to measure swim distance and distance from the bottom of the tank (Stednitz et al., 2018). To determine periods of active swimming, pauses in swimming were removed from the data set, defined as at least 20 consecutive frames (2 s total) with no between-frame speed measurement of greater than 1.5 cm/s. Fastest speed was defined as the speed during the fastest 100 consecutive frames (10 s total) within the 5-min swim test. Similar differences between genotypes were found when defining the maximum speed as the fastest 50 or 30 consecutive frames, or the average of the 30 fastest 1-s periods (Fig. S14, Table S5). Following the test period, the fish were returned to their home tanks.

### Histology

Ten-month-old WT and mutant adult zebrafish were euthanized by hypothermic shock and fixed in Bouin's solution (Sigma-Aldrich), then washed in 70% ethanol. Fixed fish were bisected parasagitally or transversely, processed for paraffin embedding, sectioned at 7 µm thickness, and stained with Hematoxylin and Eosin by a histologist from the University of Oregon Institute of Neuroscience. Muscle morphology was analyzed by a fish pathologist from the Zebrafish International Resource Center who was blinded to genotype. Differential interference contrast microscopy images of tail epaxial muscle transverse sections along the dorsal fin were taken using a Leica DMLB microscope and muscle fiber cross-sectional areas were measured using Fiji. For each genotype, five fish were sectioned and a total of 400-1000 muscle fibers from the left side epaxial muscle proximal to the spine were measured across five to six sections of each fish.

## Supplementary Material

Supplementary information
