# Supplementary material for: Zebrafish mbnl mutants model physical and molecular phenotypes of myotonic dystrophy
Source: Dis Model Mech. 2021 Jun 14;14(6):dmm045773. doi: 10.1242/dmm.045773 (PMC8246264; doi:10.1242/dmm.045773)
Supplement: Supplementary information [file dmm-14-045773-s1.pdf]

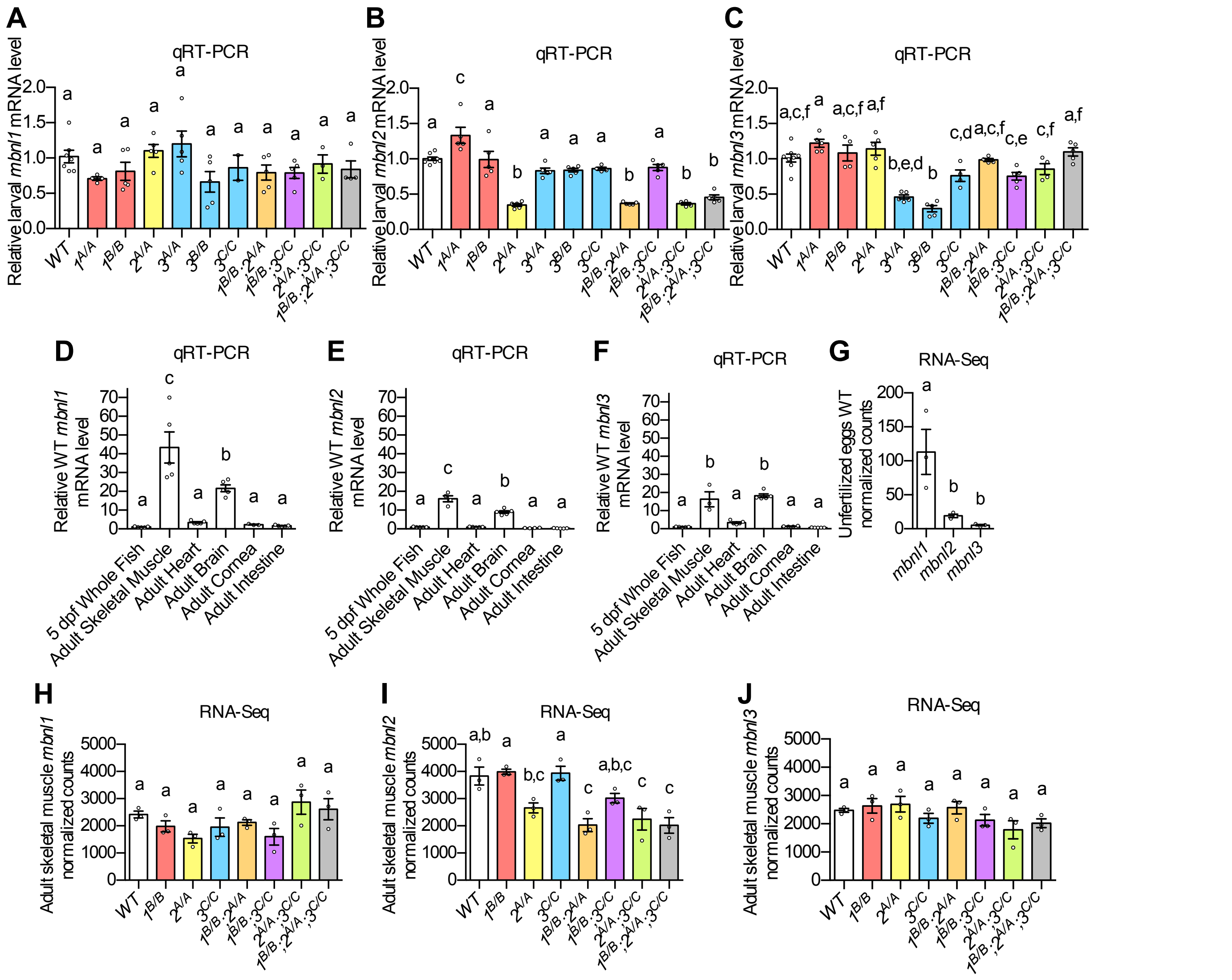

Figure S1. Effects of *mbnl* mutations on *mbnl* mRNA levels.
A-C. qRT-PCR showing the levels of (A) *mbnl1,* (B) *mbnl2,* and (C) *mbnl3* mRNAs in WT and *mbnl* mutant 5 days post fertilization whole larval zebrafish.
D-F. qRT-PCR showing relative levels of (D) *mbnl1* mRNA, (E) *mbnl2* mRNA, and (F) *mbnl3* mRNA in WT 5 dpf whole larvae, adult skeletal muscle, heart, brain, cornea, and intestine.
G-J. RNA-Seq data showing the normalized counts of WT *mbnl1*, *mbnl2*, and *mbnl3* RNAs in unfertilized eggs (from Mehjabin *et al.,* 2019), and (H) *mbnl1,* (I) *mbnl2,* and (J) *mbnl3* mRNAs in WT and *mbnl* mutant adult zebrafish skeletal muscle.
Data information: In (A-J) data are presented as mean ± SEM. Each dot represents RNA from a pool of 5 larvae or one adult fish. Data were analyzed by ordinary one-way ANOVA with Tukey's multiple comparisons test. Data bars that do not share a letter above them are significantly different from one another. Raw data and statistical analysis details are in Table S5.

Disease Models & Mechanisms • Supplementary information

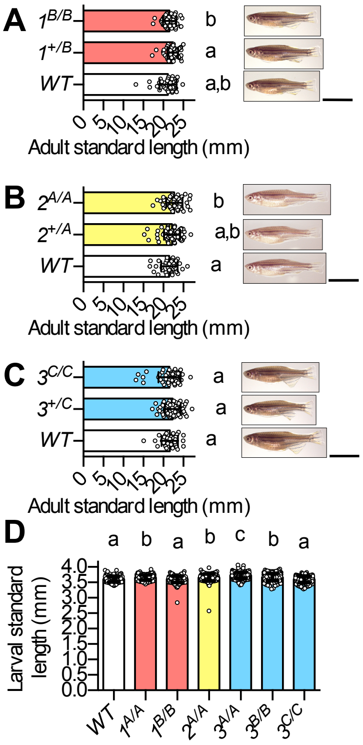

Figure S2. Single *mbnl* mutant zebrafish were not decreased in size.

A-C. Standard length of (A) 83 dpf clutchmates from an incross of $1^{+/B}$ fish, (B) 83 dpf clutchmates from an incross of $2^{+/A}$ fish, and (C) 78 dpf clutchmates from an incross of $3^{+/C}$ fish. Example images of fish of each genotype are next to each graph. Scale bar = 10 mm.

D. Standard length of 7 dpf larval fish.

Data information: In (A-D) *mbnl1* mutant alleles are denoted as 1^A and 1^B, *mbnl2* alleles as 2^A, and *mbnl3* alleles as 3^A, 3^B, and 3^C. Data are presented as mean ± standard deviation. Each dot represents one fish. Data were analyzed by ordinary one-way ANOVA with Tukey's multiple comparisons test. Data bars that do not share a letter above them are significantly different from one another. Raw data and statistical analysis details are in Table S5.

Disease Models & Mechanisms • Supplementary information

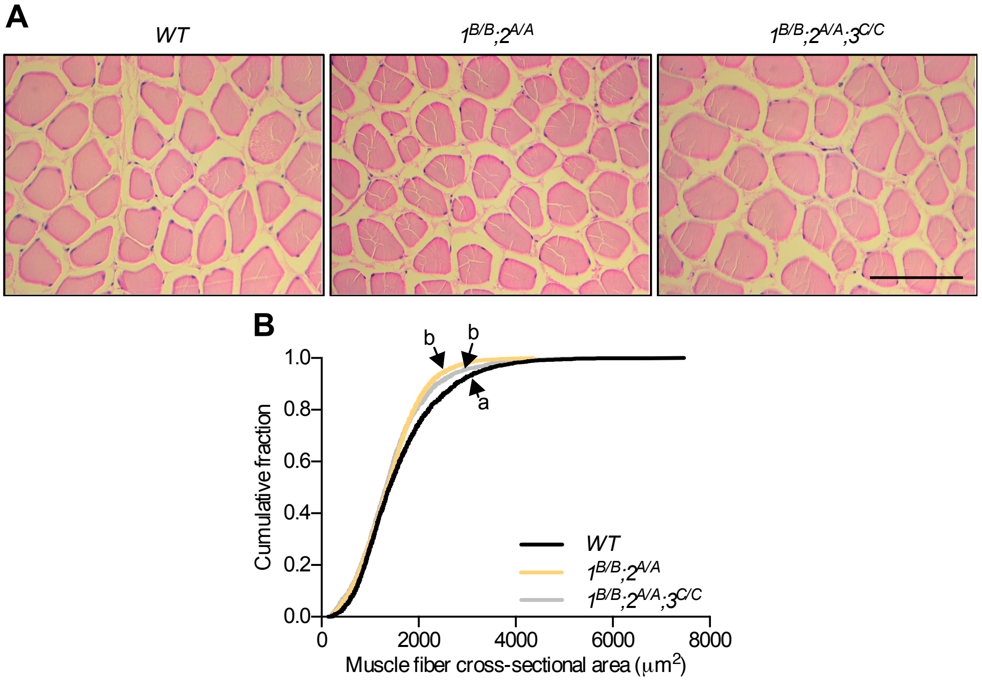

Figure S3. Adult zebrafish WT and *mbnl* mutant muscle fibers were structurally similar.
A. Representative hematoxylin and eosin stained transverse sections of epaxial muscle fibers from the tails of adult zebrafish. Scale bar = 100 μm. **B.** Distribution of muscle fiber cross-sectional areas from transverse sections of epaxial muscle fibers from the tails of adult zebrafish. Individual muscle fibers were measured from 5-6 sections each of 5 fish from each genotype. WT n=2928, $1^{B/B};2^{A/A}$ n=3819, $1^{B/B};2^{A/A};3^{C/C}$ n=2846. Data were analyzed using the Kruskal-Wallis test with Dunn's multiple comparisons test. Curves with different letters are significantly different from one another.

Zebrafish *mbnl1* exon 5 / Human *MBNL1* exon 5

```
           1                                                50
Zebrafish  gactgtatttctgcatgccttagtcctgatatccccctgtatactgcatt
Human      gccttta.ttgtgcatg.cttagtcttgttat.tcgttgtatatggcatt

           51                                              100
Zebrafish  ccaatgatttg..tttttatttgtttttttttctcacctaccc.aaaatg
Human      ccgatgatttgtttttttatttg...ttttttctcacctacccaaaaatg

           101                                             150
Zebrafish  cactgctgccccccatgatgcacctctgcttgctgtttatgttaatgcgct
Human      cactgctgccccccatgatgcacctctgcttgctgtttatgttaatgcgct

           151                                             200
Zebrafish  tgaaccccattggcccattgccatcatgtgctcgctgcctgctaattaag
Human      tgaaccccactggcccattgccatcatgtgctcgctgcctgctaattaag

           201                                             250
Zebrafish  ACTCAGTCGGCTGTCAAATCACTGAAGCGACCCCTCGAAGCCACCTTTGA
Human      ACTCAGTCGGCTGTCAAATCACTGAAGCGACCCCTCGAGGCAACCTTTGA

           251                                             300
Zebrafish  CCTGgtactatgacctttcaccttttttgcttggcatgttgctttattgta
Human      CCTGgtactatgacctttcaccttttagcttggcatgtagctttattgta

           301                                             350
Zebrafish  g..........tgtctttaacaaacaa....aaaaactgagatataatct
Human      gatacaagtttttttttta..aatcaactttaaa.....atatata....

           351                                             400
Zebrafish  gcaagcatagtccttttgtgtttgtttaatgcctctgtt...gtgttctg
Human      ..........tcctttt...............ttctgttatagagt..tg

           401                                             450
Zebrafish  t....taccatctcgtgtg.atcaactgactgtgg.....cgagcaagaa
Human      taaagtacaa.......tgaaaaaactgagtgtggtttcctga.caa.aa

           451                                          497
Zebrafish  ttgttag.cagacgccgccgcaatc...............agatcat
Human      ttagtagaaaga.....ctataatctaagtacatagatggatatcat
```

Figure S4. The introns surrounding zebrafish *mbnl1* exon 5 contain potential Mbnl protein binding sites.
Zebrafish *mbnl1* exon5 (bold upper case letters) and its surrounding intronic sequences (lower case letters) were aligned with orthologous human sequences using the EMBOSS Water program. Potential YGCY (Y=pyrimidine) Mbnl protein binding sites are indicated in red.

Disease Models & Mechanisms • Supplementary information

Zebrafish *mbnl2* exon 5 / Human *MBNL2* exon 5

```
               1                                                  50
Zebrafish      ttcgtttatgtgcatgcctattgttttgtatgtggtatactgcattcaga
Human          tttgttgttgtgcatgcctagtgttttgtacgttgtatattgcattcaga

               51                                                100
Zebrafish      ttatttttgtttttgttttttttccttctcacctatccacaatgcaatgc
Human          ata.............ttttttccttctcacctatccacaatgcaatgc

               101                                               150
Zebrafish      tgtccccatgatgcacctctgcttgctg.ttacctgtatgttaatacgct
Human          cgttcccatgatgcacctctgcttgctgtttacctgtatgttaattcgct

               151                                               200
Zebrafish      tgaatcccattggcccactgccatcatgtgctcgctgcctgctattaaaa
Human          tgaatcccattggcccactgccatcatgtgctcgctgcctgttattaaaa

               201                                               250
Zebrafish      gACTCAGTCGACTGCCAAAGCAATGAAGCGACCCCTCGAGGCATCTGTAG
Human          gACTCAGTCGACTGCCAAAGCAATGAAGCGACCTCTCGAAGCAACTGTAG

               251                                               300
Zebrafish      ATCTGgtactatgacctttcacctttttgctttgcatgtagctttttttaat
Human          ACCTGgtactttgacctttcacctttcgctttgcatgtagcttttt.....

               301                                               350
Zebrafish      gtactgtagcagaac..tcacagaaaaaaaaagtgatttaatttggcttc
Human          ......cag.agaaccatctgag.................atttg...ta

               351                                               400
Zebrafish      ttgctt.........ttgatacaaaca...................gtaa
Human          ttacttgtaaaatactggttgcaaacaatcattattattaatttatggaa

               401                                               450
Zebrafish      aaagtcaccaa....gtg.............tacttgtatgtagaactta
Human          aaaat..ccaataaggtgtttagccatctcattctcatatgta.......

               451                                         494
Zebrafish      ccatcagtcgtaattcttgaagcctagtaaacgttaagtgcctt
Human          ..atctgt.gaaataaattaatcctaacaaattttaa.tacctt
```

Figure S5. Zebrafish *mbnl2* exon 5 and its surrounding introns contain potential Mbnl protein binding sites.

Zebrafish *mbnl2* exon 5 (bold upper case letters) and its surrounding intronic sequences (lower case letters) were aligned with orthologous human sequences using the EMBOSS Water program. Potential YGCY (Y=pyrimidine) Mbnl protein binding sites are indicated in red.

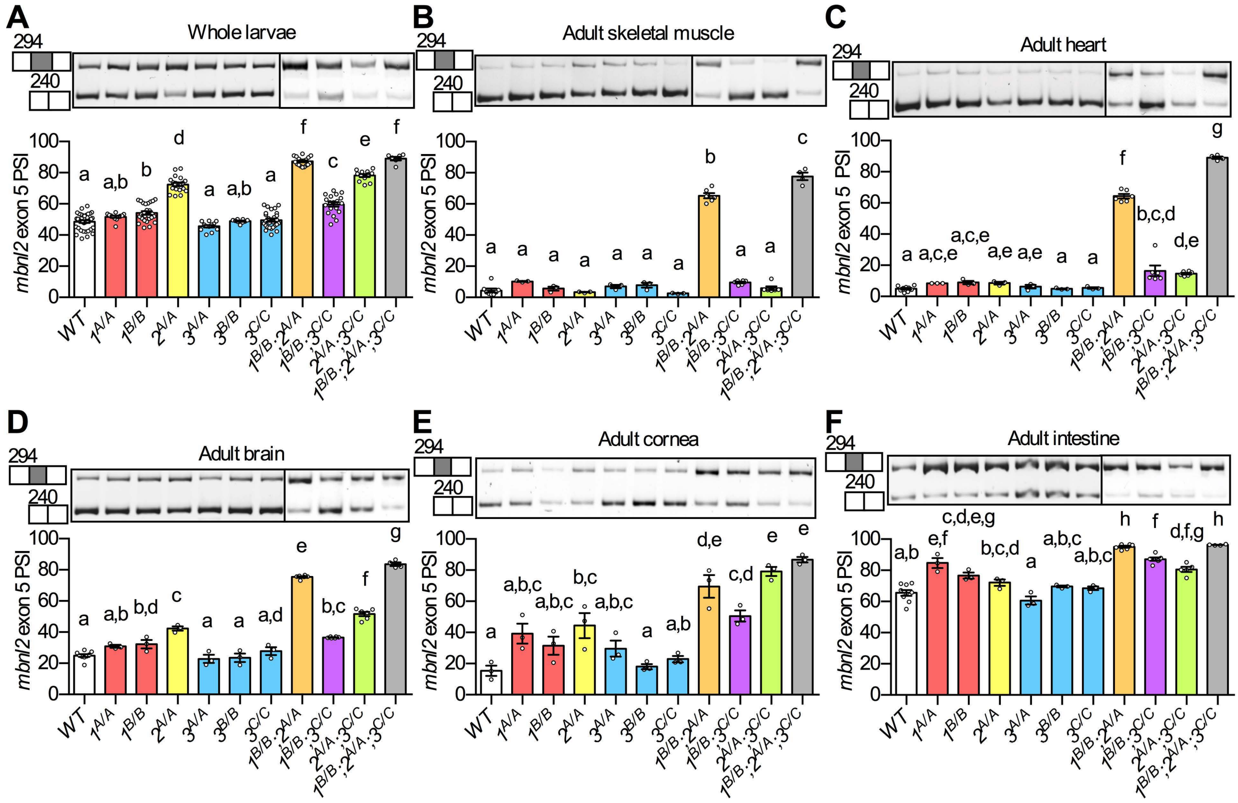

Figure S6. Alternative splicing of *mbnl2* exon 5 was misregulated across tissues in zebrafish *mbnl* mutants.

A-F. RT-PCR analysis showing percent spliced in (PSI) of *mbnl2* exon 5 in WT and *mbnl* mutant (A) whole 5 dpf larvae, and in adult (B) skeletal muscle, (C) heart, (D) brain, (E) cornea, and (F) intestine. *MBNL2* exon 5 inclusion is increased in human DM1 patients (Tabs 5A and S7A of Table S5).

Data information: In (A-F) *mbnl1* mutant alleles are denoted as 1^A and 1^B, *mbnl2* alleles as 2^A, and *mbnl3* alleles as 3^A, 3^B, and 3^C. Representative RT-PCR gels are shown above each graph with band sizes in bp shown on the left. White boxes represent constitutive exons and gray boxes represent alternative exons. Dividing lines indicate samples run on separate gels. Each dot represents RNA from one adult fish or a pool of five larvae. Data are presented as mean ± SEM. Data were analyzed by ordinary one-way ANOVA with Tukey's multiple comparisons test. Data bars that do not share a letter above them are significantly different from one another. Raw data and statistical analysis details are in Table S5.

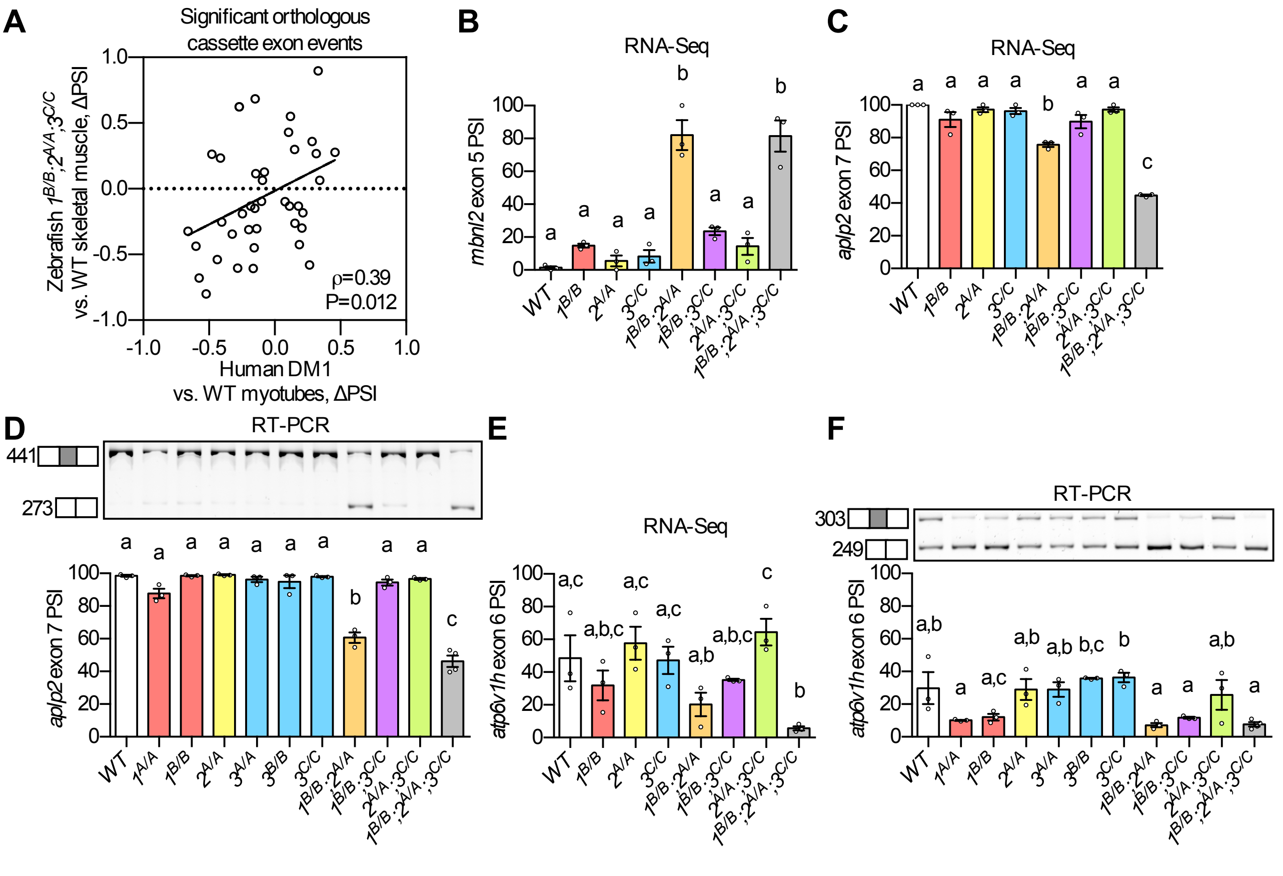

Figure S7. Misregulation of many DM-associated alternative splicing events was conserved in zebrafish *mbnl* mutants.

A. Change in percent spliced in values (ΔPSI) between mutant and WT are shown for orthologous exons in zebrafish $1^{B/B};2^{A/A};3^{C/C}$ skeletal muscle and in human DM1 patient-derived myotubes.

B-F. (B,C,E) RNA-Seq and (D,F) RT-PCR analyses showing PSI of (B) *mbnl2* exon 5, (C-D) *aplp2* exon 7, and (E-F) *atp6v1h* exon 6 in WT and *mbnl* mutant adult zebrafish skeletal muscle. Orthologous human exon inclusion in DM patient tissues is increased for *MBNL2* and decreased for *APLP2* and *ATP6V1H* (Tabs 5A and S7A of Table S5).

Data information: In (A) ρ is the Spearman's rank correlation coefficient. In (A-F) homozygous *mbnl1* mutant alleles are denoted as 1^A and 1^B, *mbnl2* alleles as 2^A, and *mbnl3* alleles as 3^A, 3^B, and 3^C. Representative RT-PCR gels are shown above each graph with band sizes in bp shown on the left. White boxes represent constitutive exons and gray boxes represent alternative exons. Each dot represents RNA from one fish. Data are presented as mean ± s.e.m. Raw data and statistical analysis details are in Table S5.

Disease Models & Mechanisms • Supplementary information

Zebrafish *aplp2* exon 7 / Human *APLP2* exon 7

```
           1                                                 50
Zebrafish  taaa.....atac..........ag.aaatagt.ctcagc..........
Human      taaatgtctctactctgtggggaagcaacttgtcctcagcaaggctggcc

           51                                               100
Zebrafish  ........agctt.......ccggttatgcagactttcatatttttgta
Human      tgagctggagctttcggccaccgggcctccaggct...............

           101                                              150
Zebrafish  acaaagatcttcaagtttaatatctatggagaaagtgtttacaaaaactt
Human      .......ccgtccag.......tctcagg.................cctc

           151                                              200
Zebrafish  ctgca........cccag......agtgtctgttgcgctatatattagaa
Human      ccccagcccatcccccagctcgcca..gcctgtagc.....at.tttgaa

           201                                              250
Zebrafish  ggtattgg.......tgccccgtgtggctg..gaggctctaactgcgcct
Human      .gcatttgacgtcactgcctc.tgt.cctgctgacactctgac.......

           251                                              300
Zebrafish  cctcctgtgtt......agCTGTGTGTTCTCTGGAGGCGGAGACCGGCCC
Human      ...cat...tttcacacagCTGTCTGCTCCCAGGAGGCGATGACGGGGCC

           301                                              350
Zebrafish  CTGCCGCGCCTCTATGCCTCGCTGGCACTTCGAC..ATGCAGCAGAGGAA
Human      CTGCCGGGCCGTGATGCCTCGTTGGTACTTCGACCTCTCCA..AGGGAAA

           351                                              400
Zebrafish  GTGTGTGCGCTTCATCTACGGAGGCTGCGCCGGCAACCGCAACAATTTCG
Human      GTGCGTGCGCTTTATATATGGTGGCTGCGGCGGCAACAGGAACAATTTTG

           401                                              450
Zebrafish  ACTCAGAGGAGTACTGCATGGTCGTGTG.CAAGCGCCTGAgtaagtcac.
Human      AGTCTGAGGATTATTGTATGGCTGTGTGTAAAGCG.ATGAgtaagtc.ct

           451                                              500
Zebrafish  .actcgc..........tactgaagactccgccccttctg..ctccgcct
Human      gcctcgcgctggtcccgtgcggcag.caccgtcctgtctggcgtccgtct

           501                                              550
Zebrafish  tcct.............ctgc.tccgt.......cct...........t
Human      ccctgccgtcttcgtggctgcatctgtgtggtgtccctgcccactcgggt

           551                                              600
Zebrafish  ttttgct.....ccgccttcctctgctc....tgccctct.....tatgc
Human      gtttgctgtcggtcgtcttcccctcatctttgtgctttctagatctaggc

           601                                  641
Zebrafish  tctgc.ctccttctgctctgtcacagtgctaa.ctctgttc
Human      tttgctctcctgccg.......gcagtggtaagctcagttc
```

Figure S8. Zebrafish *aplp2* exon 7 and its surrounding introns contain potential Mbnl protein binding sites.
Zebrafish *aplp2* exon 7 (bold upper case letters) and its surrounding intronic sequences (lower case letters) were aligned with orthologous human sequences using the EMBOSS Water program. Potential YGCY (Y=pyrimidine) Mbnl protein binding sites are indicated in red.

Disease Models & Mechanisms • Supplementary information

Zebrafish *atp6v1h* exon 6 / Human *ATP6V1H* exon 6

```
               1                                                 50
Zebrafish      agactgaaa.........taaattcaaggcca..ctctcgcacagattt
Human          agaatgtaacgatatttagaacattttatgtcatccccttgc.....ttt

               51                                               100
Zebrafish      ttagacttttgcctg.tcaagaacaaactgtctctctcatgcgggagtca
Human          tta...tttagtaggattaggaataaa........attatagggaaaaa

               101                                              150
Zebrafish      tctgtcttcctgccaccattgtccaa.....gcacacg........gctc
Human          t...tatt.......acatag...aattaggggaaaagtttgcttagctc

               151                                              200
Zebrafish      actttt....gct................tcaaacgaggccca.....a
Human          ccttttgtaagctgatacattaggataaaatcaaa......acaaaacta

               201                                              250
Zebrafish      aatcaccgttttttttctttctatctttttttttgttgttgt..tagAAAC
Human          aataatgagttatttatttc...................gtaatagAAAC

               251                                              300
Zebrafish      TACATGGTACAGGTGCTGAACCTGGAACAGGGACCATCTCCCCCAGTGAA
Human          TGCGTGGTAGCGGTGTTGCTGTTGAAACAGGAACAGTCTCTTCAAGTGAT

               301                                              350
Zebrafish      gtgagtat.....gtcacagatgaaggaggcagatgtgcagctgtgtgaa
Human          gtaagtatattccttcactg......................gcctaaa

               351                                              400
Zebrafish      cgagcttccgtcccc..........tttcagaggctgag..atcttcac
Human          ........catccccaaaataaattattt.......tgagtcatataca.

               401                                              450
Zebrafish      ccctctctt....ttttgagatgcagcacatgctgttt.cttgtccactg
Human          ......cttaaaattttg..........aaggtgtttactag.......

               451                                              500
Zebrafish      cttttcccc.........cccat..tcatatccact...gtgaaaaataa
Human          ...ttccccaactataaatccatggtcacctccagttaggttaa......

               501                                              550
Zebrafish      gcatttttattacaaa.................ggtc............
Human          ....ttgtattataaactatatataaatattgatggtctgtggagatcat

               551                     576
Zebrafish      ctttcctct.............cttg
Human          cttttctgtggtagacataagacttg
```

Figure S9. Zebrafish *atp6v1h* exon 6 and its surrounding introns contain potential Mbnl protein binding sites.

Zebrafish *atp6v1h* exon 6 (bold upper case letters) and its surrounding intronic sequences (lower case letters) were aligned with orthologous human sequences using the EMBOSS Water program. Potential YGCY (Y=pyrimidine) Mbnl protein binding sites are indicated in red.

Disease Models & Mechanisms • Supplementary information

Zebrafish *atp2a1l* exon 23 / Human *ATP2A1* exon 22

```
                1                                                 50
Zebrafish    ~~~~~~~~~~~~~~~~~~~ttctaaaca..tgaaccttagtgtatgtttt
Human        ccgccccgccccgccccgtactttgcaggtg.........gtaagttt.

                51                                               100
Zebrafish    atataatgttttcctttttaatatacagtatattaattgca....ctttt
Human        .............ct..........cag.....ccctggcaggacctgtg

                101                                              150
Zebrafish    t........ttcctcat..............acaact.............
Human        tccgccccgttccccctgcgcctgcaggggccacatctccggggcagccc

                151                                              200
Zebrafish    ...tgtttcaactaaaaagagtgtttttaatcag.......atctaattc
Human        cactgcctc....................ctcagcccccacagc....cc

                201                                              250
Zebrafish    ctata.ccccttttcttatctttctca.ctcatttaattgatttcccact
Human        ctatagcccccatgc..........cacctccctgccttga........t

                251                                              300
Zebrafish    aaccaa....ctatattcatttctag~~~~~~~~TCTAAACAGTTCTCAC
Human        aac..agtgcctcttgtcctctctggccatagGAT...AACTGTTC.CCC

                301                                              350
Zebrafish    CTCC.......CAGATTTCAAGTAACAGAAGCAAGgtatacaacacccca
Human        CTCCTCCATCTCTGAGCCCGTGTCAC.......AGgtat....cacccc.

                351                                              400
Zebrafish    gctgctt..............ttctgtg..tctg..............tc
Human        .cttcttgccctcagcccagctgctgtgcccctgccacccgcgccccctc

                401                                              450
Zebrafish    tg.....tacatgtc...tac.aggtttcaatccttttg..........c
Human        agccccttgcgcgtcgcatccaagg..tca...cttgtgctcgcagctcc

                451                                              500
Zebrafish    atgtttagtggttag..attgatgttgct....tt.........gtgg..
Human        acctggagccgtt.gccactgctgctgctgcgcttccagtcagggtgggc

                501                                              550
Zebrafish    ........tcttaaaccac......tctgtttttagct...atgcatgaa
Human        cgctggcctc.....ccactgggcgtcag..tttggctcccaggc.....


                551                                              600
Zebrafish    gcgcttgtgaaagttatacttaattatacttcttaatggcaagagcttta
Human        ...cctgggcag~~~~~~~~~~~~~~~~~~~~~~~~~~~~~~~~~~~~~~

                601              618
Zebrafish    tctagttcataatttgca
Human        ~~~~~~~~~~~~~~~~~~
```

Figure S10. The introns surrounding zebrafish *atp2a1l* exon 23 contain potential Mbnl protein binding sites.

Zebrafish *atp2a1l* exon 23 (bold upper case letters) and its surrounding intronic sequences (lower case letters) were aligned with orthologous human sequences using the EMBOSS Needle program. Potential YGCY (Y=pyrimidine) Mbnl protein binding sites are indicated in red.

Disease Models & Mechanisms • Supplementary information

Zebrafish *ryr1b* exon 72 / Human *RYR1* exon 70

```
           1                                                50
Zebrafish  actccatttgt......tcagacaattgctgacc...........tcat
Human      actgcccttctcaggtctcagagaa....cgaccccccaccccgagccaa

           51                                              100
Zebrafish  gtcctgttgaggac.....agacactgtttctgctctcaca.ggcggtt.
Human      ggcctggaaatgcccagctagagaat...........acatggcgggtg

           101                                             150
Zebrafish  ...tatagg........gctg.....ttgatgtttttatttatttattt
Human      gggcagaggaggtggggtgctggcaacttggag................

           151                                             200
Zebrafish  atttatttgtcctg....tctgtgagggtgtaagttttaacagtgc.tgt
Human      .....ttgggcctgggcttctctgcggggct..ggggtaacccttcttgt

           201                                             250
Zebrafish  ctctctctg.tgtcctgtcttggtgtggattcggggcctgtagGCCGGAG
Human      ctctgtctgcggtcc......ggtg...........aagcagGCGGGAG

           251                                             300
Zebrafish  ATGGAGAGgtcagaccgtgtttttgcacgataaaaaaaagagctgtg..c
Human      ATATACAGgtcagccc........cac...........atctgggacc

           301                                             350
Zebrafish  tgat.cgcatggcgcagaatgttgggtttt....tcatcaaactccca..
Human      t..tccgcatgtc......tcttggctaatgccctctt....cccccagc

           351                                             400
Zebrafish  .....cacacactggcc.......aa....tgct..........cacac
Human      ctctgcacgccccccgcctcgagaaaacccctgcttctgttccccaccccc

           401                                             450
Zebrafish  atcatacacacaaaagtccaa.........accctcattcactgccagt
Human      gtcctcccc.......tcccagcccccacccatcctccttcccttcc...

           451                                             500
Zebrafish  gtgtgtgaggtctgttgttgcct...............cgtt.tgatga
Human      ...............cttccctccccaccaccccccatccgttcccatcc

           501              521
Zebrafish  aagccctcagccatagacttt
Human      caaccctcag.......cttt
```

Figure S11. The introns surrounding zebrafish *ryr1b* exon 72 contain potential Mbnl protein binding sites.

Zebrafish *ryr1b* exon 72 (bold upper case letters) and its surrounding intronic sequences (lower case letters) were aligned with orthologous human sequences using the EMBOSS Water program. Potential YGCY (Y=pyrimidine) Mbnl protein binding sites are indicated in red.

Disease Models & Mechanisms • Supplementary information

Zebrafish *ank3b* exon 36 / Human *ANK3* exon 35

```
           1                                                  50
Zebrafish  ggtgcacag.gcttgtgcttttttttcagcagcttctgtggttgcggcttt
Human      gatgtacagttcttgatatttttt...............gtt..ggcttt

           51                                                100
Zebrafish  tttcttttcatttg..ttgcccctttatgttcct...tca.ctgtggctt
Human      tt..ttaaaatttgctttgc.........tttctaaatcagct.tgcatt

           101                                               150
Zebrafish  tgcggt...aacacaatcgcctttgttcacatcctgatgtt.tcttcccc
Human      tgtgctccctactcagt..cctt..ttcatatcctgatattctttt...c

           151                                               200
Zebrafish  cctgctcttcattacgttatcccctctgtttctgcaatgcatcttgggaa
Human      cctgctgtccattataatattcc..ttgtctctgcaatgcatctt.ggaa

           201                                               250
Zebrafish  ccaaaagGATGTGGATTCAGATCCCGAGGAAGAGgtaatagtgctgtccc
Human      ccaaaagGAGACAGAGTCAGATCAAGATGATGAGgtaata..........

           251                                               300
Zebrafish  ct...catttccatgtggtgtgctagttgctttagctct..........g
Human      .taaacacttc................tgcttttattctatcagaagtag

           301                                               350
Zebrafish  gattg..attg..tttgt..........tttaaggcggctttaacatgag
Human      aatagaaattgaatctgtatgacagaagttgcagagggc..taaagtgtg

           351                                               400
Zebrafish  tacacatacag..aacacatacagcatgctgctgcaattttctg....ca
Human      ..........ggaaacatataaattat........atttttcaggtctta

           401                                               450
Zebrafish  agttg..............attcttt..........ttcactcatccatg
Human      agtaggtaaaagggtatatatt..tttcttaaatggttcactc.ttcttc

           451                                     490
Zebrafish  cgcaagtattgttgcctggactgtcgag.acttaaa.ata
Human      cacacgt............acttaagagtacttaaatata
```

Figure S12. The introns surrounding zebrafish *ank3b* exon 36 contain potential Mbnl protein binding sites.
Zebrafish *ank3b* exon 36 (bold upper case letters) and its surrounding intronic sequences (lower case letters) were aligned with orthologous human sequences using the EMBOSS Water program. Potential YGCY (Y=pyrimidine) Mbnl protein binding sites are indicated in red.

Disease Models & Mechanisms • Supplementary information

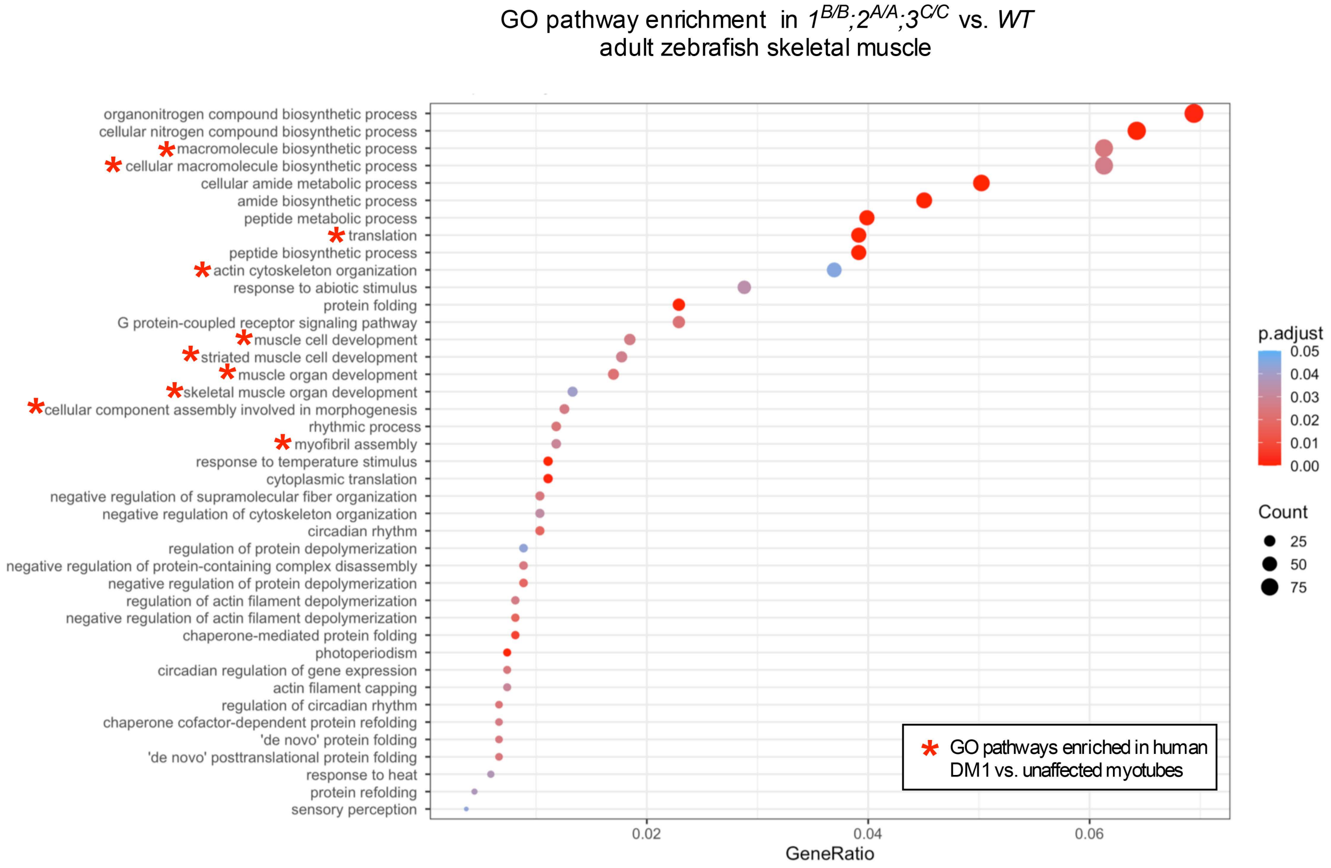

Figure S13. Differentially expressed genes in zebrafish *mbnl* mutants and human DM1 myotubes belong to overlapping gene ontology pathways.

Significantly enriched gene ontology (GO) pathways for genes that were differentially expressed in $1^{B/B}$; $2^{A/A}$;$3^{C/C}$ vs. WT adult zebrafish skeletal muscle. Red asterisks indicate those GO pathways that were also enriched in differentially expressed genes between human DM1 vs. WT myotubes. See Table S6 for complete lists of differentially expressed genes and enriched GO pathways in zebrafish and human. Data information: p. adjust indicates adjusted p-value. Count indicates number of differentially expressed genes belonging to each GO pathway. Raw data are in Table S6.

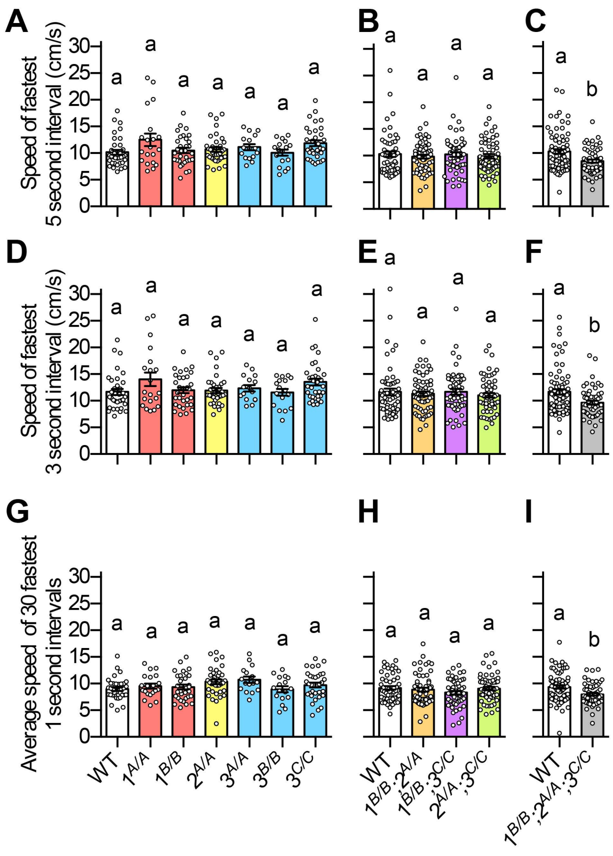

Figure S14. Zebrafish triple homozygous *mbnl* mutants swim at decreased speed. This figure contains additional analysis of data from the five minute swim test in Fig. 2.

A-C. Speed in cm/s of fastest five second interval within the five-minute swim test for (A) single, (B) double, and (C) triple homozygous *mbnl* mutant fish.

D-F. Speed in cm/s of fastest three second interval within the five-minute swim test for (D) single, (E) double, and (F) triple homozygous *mbnl* mutant fish.

G-H. Average speed in cm/s of 30 fastest one second intervals within the five-minute swim test for (G) single, (H) double, and (I) triple homozygous *mbnl* mutant fish.

Data information: *mbnl1* mutant alleles are denoted as 1^A and 1^B, *mbnl2* alleles as 2^A, and *mbnl3* alleles as 3^A, 3^B, and 3^C. In (A-I) each dot represents one fish and data are presented as mean ± s.e.m. In (A,B,D,E,G,H) data were analyzed by ordinary one-way ANOVA with Tukey's multiple comparisons test and in (C,F,I) data were analyzed by an unpaired Student's t-test. Data bars that do not share the same letter above them are significantly different from one another. Raw data and statistical analysis details are in Table S5.

Disease Models & Mechanisms • Supplementary information

Table S1. Primers used in this study.

Click here to download Table S1

Table S2. Zebrafish *mbnl* mutant sequences.

For each zebrafish *mbnl* mutant used in this study, this table lists the University of Oregon allele number, exon that was mutated within the target gene, guide RNA (gRNA) used to generate the mutant, forward primers, reverse primers, and restriction sites used to genotype the mutants by restriction fragment length polymorphism, ZFIN Gene ID for the targeted gene, GRCz11 genome coordinates and coding sequence of the targeted exon, and predicted protein coding sequence. Sequences that are changed compared to WT are indicated in bold. Sequences of primers from this table are listed in Table S1.

Click here to download Table S2

Table S3. Zebrafish single, double, and triple homozygous *mbnl* mutants are viable to adulthood.

For each zebrafish single, double, and triple homozygous mutant *mbnl* genotype, this table lists the cross that was used to generate the genotype, the percent of adult offspring expected of the genotype according to Mendelian genetics, and the actual percent and number of adult offspring observed with the genotype.

Click here to download Table S3

Table S4. Significantly misregulated alternative splicing events identified by RNA-Seq in zebrafish *mbnl* mutants.

Each tab contains a list of alternative splicing events (cassette exon, mutually exclusive exon, retained intron, alternative 5' splice site, or alternative 3' splice site) that were identified by RNA -Seq analysis as being significantly misregulated in adult zebrafish *mbnl* mutant skeletal muscle compared to WT. All genome coordinates are from the GRCz11 genome assembly, except for WT vs. 1B/B;2A/A;3C/C, for which a list of significantly misregulated cassette exons identified from a preliminary analysis of the GRCz10 genome assembly is also shown. Complete RNA-Seq data are available from NCBI GEO #GSE145270.

Click here to download Table S4

Disease Models & Mechanisms • Supplementary information

Table S5. Source data and statistical analyses for figures.

Each tab contains the source data and details of statistical analysis for the indicated figure.

Click here to download Table S5

Table S6. Differential gene expression and gene ontology (GO) analysis of zebrafish adult skeletal muscle WT vs. 1B/B;2A/A;3C/C and unaffected vs. DM1 human myotubes

The first two tables contain lists of genes that were significantly differentially expressed in WT vs. 1B/B;2A/A;3F/F adult zebrafish skeletal muscle or in unaffected vs. DM1 human myotubes. The second two tables contain lists of significantly enriched GO terms for the differentially expressed zebrafish or human genes.

Click here to download Table S6

Disease Models & Mechanisms • Supplementary information
